# Cold-priming causes dampening of oxylipin biosynthesis and signalling during the early cold- and light-triggering response of *Arabidopsis thaliana*

**DOI:** 10.1093/jxb/erab314

**Published:** 2021-06-29

**Authors:** Andras Bittner, Bettina Hause, Margarete Baier

**Affiliations:** 1 Plant Physiology, Freie Universität Berlin, Dahlem Centre of Plant Sciences, Königin-Luise-Straße 12–16, 14195 Berlin, Germany; 2 Leibniz Institute of Plant Biochemistry, Weinberg 3, 06120 Halle, Germany; 3 University of Birmingham, UK

**Keywords:** Cold, high light, jasmonic acid, memory, OPDA, oxylipin, plastid signalling, priming, *tAPX*, *ZAT10*

## Abstract

Cold-priming uncouples cold and light regulation of otherwise tightly co-regulated genes. In this study, we focused on the early regulatory processes in Arabidopsis within the first 2 h in cold and in high light after a 5-d lag-phase at 20 °C and 24 h cold-priming at 4 °C. Priming quickly modified gene expression in a trigger-specific manner. In the early stress-response phase during cold and high-light triggering, it reduced the regulatory amplitudes of many up- and down-regulated genes. A third of the priming-regulated genes were jasmonate-sensitive, including the full set of genes required for oxylipin biosynthesis. Analysis of wild-type and mutant plants based on qPCR demonstrated that biosynthesis of the jasmonic acid (JA) precursor 12-oxo phytenoic acid (OPDA) relative to the availability of JA dampened the response of the genes for oxylipin biosynthesis. In oxylipin biosynthetic mutants, cold-priming more strongly affected genes involved in the biosynthesis of OPDA than in its conversion to JA. In addition, priming-dependent dampening of the triggering response was more linked to OPDA than to regulation of the JA concentration. Spray application of OPDA prior to triggering counteracted the priming effect. Regulation of the oxylipin hub was controlled by modulation of the oxylipin-sensitivity of the genes for OPDA biosynthesis, but it was insensitive to priming-induced accumulation of thylakoid ascorbate peroxidase, thus identifying a parallel-acting cold-priming pathway.

## Teaser

Short cold-priming in Arabidopsis dampens subsequent responses to cold and high light by modulating the oxylipin sensitivity of genes associated with oxylipin biosynthesis and by attenuating OPDA biosynthesis relative to jasmonic acid availability.

## Introduction

Priming is a highly versatile process that prepares plants to respond more effectively to changes in conditions, whether by making the response greater or faster, or enabling a more specific response to occur (Crisp *et al.*, 2016; Hilker *et al.*, 2016; Avramova, 2019; Baier *et al.*, 2019). The priming memory is imprinted by the experience of stress and stores information for days under non-stress conditions (Ding *et al.*, 2013; van Buer *et al.*, 2019). In one of the early studies on cold-priming of Arabidopsis in the vegetative stage, priming was still referred to as ‘cold acclimation’ (Byun *et al.*, 2014); however, acclimation (according to the current definition) is a process of sequential adjustment of gene expression and physiological processes to an enduring stimulus. Byun *et al.* (2014) showed that genes that respond to 24 h at 0 °C return to pre-stress levels of expression within hours after transfer to 23 °C. Application of a second (triggering) stimulus of 0 °C after a lag-phase (memory phase) of 72 h at 23 °C then results in an improved tolerance to 2 h at –2 °C, and causes greater cold-induction of genes involved in dehydration stress control, calcium signalling, chloroplast protein synthesis, and lipid metabolism.

Subsequent studies have shown that a 24-h temperature shift from 20 °C to 4 °C under short-day conditions does not support cold-activation of *CBF*s (encoding C-repeat binding factors) or their target genes in Arabidopsis upon cold-triggering at 4 °C after a lag-phase of 5 d at 20 °C in 4-week-old plants (van Buer *et al.*, 2016), but it does result in higher expression of defence-regulated genes (Bittner *et al.*, 2020) and in decreased susceptibility to pathogens (Griebel *et al.*, 2020, Preprint). The same genes are not induced in cold-primed plants by heat-filtered, high-light conditions, and some are even inversely regulated by cold-priming in response to the high light, for example the pathogen responsive genes *PR4* (At3g04720) and *PCC1* (At3g22231) (Bittner *et al.*, 2020). Other genes are only priming-sensitive in the cold, but not in the light, for example the light- and cold-inducible genes for the stress-signalling mediating zinc-finger transcription factors ZAT6 (At5g04340), ZAT10 (At1g27730; *STZ*), and ZAT12 (At5g59820) (Bittner *et al.*, 2020). Although epigenetic regulation by DNA and/or histone acetylation or methylation can mediate priming (Hilker *et al.*, 2016; Avramova, 2019; Baier *et al.*, 2019; Friedrich *et al.*, 2019), the majority of shifts in gene expression after short abiotic priming events result from transcriptional regulation, for example after 2 h of dehydration stress, 60 min of excess light, or 24 h of cold (Ding *et al.*, 2013; Ganguly *et al.*, 2019; van Buer *et al.*, 2019; Bittner *et al.*, 2020). Comparisons of the light and cold responses after cold-priming of plants has demonstrated that priming uncouples otherwise tightly linked transcriptional regulation (Bittner *et al.*, 2020). Based on results such as these, we have hypothesized that the priming memory integrates into trigger-specific signalling and modifies it (Bittner *et al.*, 2020).

To gain further insights into the early mechanisms of priming-dependent regulation of gene expression, we have refined our previous experiments and investigated the impact of cold-priming on regulation of gene expression by cold and heat-filtered, high light across 2-h time-courses. We show that the priming memory quickly dampens the response of genes that are essential for activation of biosynthesis of the jasmonic acid precursor oxylipin under stressful conditions and causes trigger-specific regulation of gene expression.

## Materials and methods

### Plant material and growth conditions

**Fig. 1.**
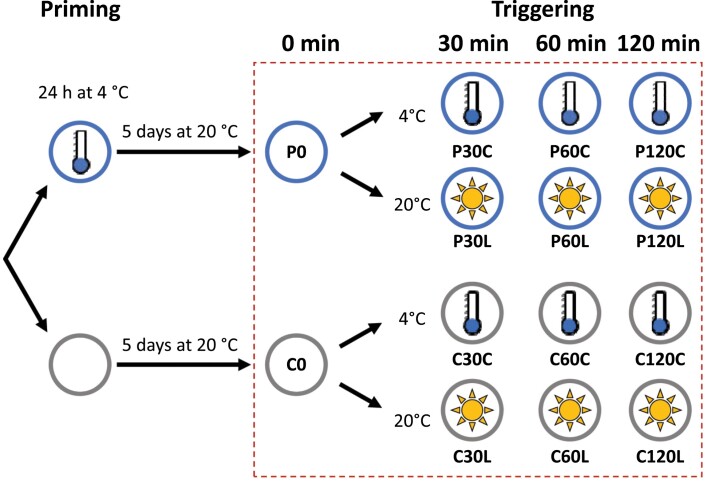
Schematic diagram of the experimental set-up. At 28 d old, half the plants were cold-primed for 24 h at 4 °C. All plants were then grown for a further 5 d under normal conditions (lag phase; 20 °C, 100–110 µmol m^-2^ s^-1^). Entire rosettes of primed (P) and control (C) plants were sampled for RNA-sequencing immediately before the start of the triggering treatments (0 min). The remaining plants were then subjected to either cold-triggering at 4 °C and 100 µmol quanta m^–2^ s^–1^ (C suffix) or light-triggering at 20 °C and 800 µmol m^–2^ s^–1^ using heat-filtered light (L suffix) and samples were taken at 30, 60, and 120 min.

In this study we used *Arabidopsis thaliana* Col-0 (N1092; obtained from the Nottingham Arabidopsis Stock Centre) and lines of *AOS*-knockout (KO; SALK_017756; Matschi *et al.*, 2015), *OPR3*-KO (SALK_201355; obtained from the Nottingham Arabidopsis Stock Centre), thylakoid ascorbate peroxidase (*tAPX*)-KO (SALK_027804), the estradiol-inducible *tAPX*-iRNAi (inducible RNAi), and the estradiol-inducible *tAPX*-iOE (inducible overexpression), which have previously been described by van Buer *et al.* (2019). Plants were cultivated in a randomized design in individual pots containing standardized soil made up of 70 volumes ‘Topferde’ (Einheitserde, Sinntal-Altengronau, Germany), 70 volumes ‘Pikiererde’ (Einheitserde, Sinntal-Altengronau, Germany), 25 volumes Perligran Classic (Knauf, Germany) and supplemented with 0.5 g l^−1^ dolomite lime (Deutsche Raiffeisen-Warenzentrale, Germany). They were grown under a regime of 10/14 h light/dark at 20±2 °C and a photon flux density of 100–110 μmol m^−2^ s^−1^ (identical to the conditions used by Bittner *et al.*, 2020). For priming, at 2.5 h after the onset of the light period, 28-d-old plants were transferred to a cold chamber at 4±2 °C under identical light conditions. The distance between the plants and the neon tubes was adjusted throughout the experiment in order to maintain the same light intensity. In common with our previous study (Bittner *et al.*, 2020), the temperature sensor that controlled the chamber settings was placed at the height of the plants. The actual leaf top temperature was monitored with an infrared thermometer. After exactly 24 h of priming, the plants were quickly transferred back to the chamber at 20 °C and randomized with the non-primed plants. After 5 d, and again at 2.5 h after the onset of the light period, 20% of the primed and 20% of the non-primed plants were transferred back to the cold chamber at 4 °C for cold-triggering (Fig. 1). The same numbers of plants were exposed to 20 °C under heat-filtered, high light with a photon flux density of 800 µmol m^–2^ s^–1^ (R7-s 500 W, Emil Lux GmbH Wermelskirchen, Germany) for light-triggering, as described previously (Bittner *et al.*, 2020. Entire rosettes of plants were sampled for RNA-sequencing (RNA-seq) at 0, 30, 60, and 120 min of the triggering treatments.

The SALK lines and the estradiol-inducible lines were tested using PCR for identity and homozygosity prior to use (Supplementary Table S1). The *OPR3* T-DNA insertion line was additionally tested using quantitative (q)PCR for the effect of the insertion on the transcript level (Supplementary Fig. S1) as no such data were available from previous studies.

### Isolation of RNA, and cDNA library construction and sequencing

Complete rosettes of five plants were harvested and pooled to form one replicate, and immediately frozen in liquid nitrogen. RNA was extracted from 100 mg mixed and ground plant material using a Gene Matrix Universal RNA Purification Kit (EURx, Gdansk, Poland) combined with a DNase treatment. After dissolving the RNA in RNAse-free H_2_O, the integrity was assessed electrophoretically on a 2% (w/v) agarose gel supplemented with 1% (v/v) formaldehyde.

For preparation of the cDNA libraries, mRNAs were enriched using Oligo(dT) magnetic beads and depleted for rRNA according to the standard procedures of the Beijing Genomic Institute (BGI, Beijing, China). Random cDNA fragments were synthesized by reverse transcription and amplified with random hexamer primers. The double-stranded cDNAs were 5´-end repaired and 3´- poly-A-tailed to enable hybridization with an oligo(dT) adapter. Following amplification of the adapter-linked DNA with adapter-specific primers, the cDNAs were sequenced by the BGI institute using a BGI-Seq-500 platform (paired-end, 100 bp length).

### Data processing

Reads with more than 5% unknown bases or with less than 80% of high-quality bases were removed from the primary data set. The remaining ‘clean reads’ were aligned to the TAIR10 reference genome using Bowtie2 (v.2.2; Langmead and Salzberg, 2012) and normalized against the total number of reads and the gene lengths (fragments per kilobase of exon per million reads mapped, FPKM) using the RSEM package (version 1.2.12).

To enable normalization of the data, the FPKM threshold in C0 and P0 plants (Fig. 1) was set to at least 0.5. In addition, genes that were generally weakly expressed were filtered out by removing those with a mean FPKM <2. The threshold for minimal response to the triggering stimulus and the minimal priming effect during triggering was set to 1.5. Finally, we removed any remaining genes for miscellaneous RNAs, RNAs representing uncharacterized or hypothetical proteins, and non-coding RNAs.

### Analysis of the RNA-seq data

We used the beta-version of ClustVis (Metsalu and Vilo, 2015) to perform principal component analysis by single-value decomposition after unit-variance scaling of the non-transformed FPKM values, heatmapping with row-centred colouring, and hierarchical clustering of the FPKM values normalized to the C0 treatment value based on Pearson confidence combined with pairwise distance comparisons. All additional heatmaps were generated with C0-normalized data in Microsoft Excel using a bidirectional symmetric colouring scheme centred in white on 0.

Hierarchical clustering of the enriched gene ontology (GO) annotations was performed using ShinyGO (Ge *et al.*, 2020), which uses the annotations of Ensembl and STRING-db, with thresholds for the error probability according to Fisher’s’ *t*-test of <0.05 and a false-discovery rate (FDR) of <0.01.

Gene groups were analysed for overlaps using the online tool for drawing Venn diagrams offered by the University of Gent (http://bioinformatics.psb.ugent.be/webtools/Venn/).

### Analysis of transcript levels by qPCR

Complete rosettes of five plants were harvested and pooled to form one replicate, and immediately frozen in liquid nitrogen. For each replicate, RNA was extracted as described above and transcribed into cDNA using Oligo(dT) primers combined with random hexamer primers. For all treatments, at least three biological replicates were analysed. Reverse transcription was carried out for 2 h at 37 °C using a High Capacity Reverse Transcription Kit (Applied Biosystems). The reaction was stopped by 5 min incubation at 85 °C. qPCR was performed for each sample with three technical replicates on a CFX96 real-time system (Bio-Rad) with 50 ng template cDNA. The amplification process was monitored fluorometrically with SYBR Green (Sigma-Aldrich). The qualities of the primer-specific reaction mixes and qPCR runs were evaluated based on the *C*_T_ values for reference samples. All transcript levels were normalized against the geometric mean of the transcript abundances of the reference genes *YLS8* (At5g08290) and *CYP5* (At2g29960). Where applicable, all primers were designed to span exon–intron borders using the QUANTPRIME software (Arvidsson *et al.*, 2008). Their specificities were assessed based on the melting curves after 40 cycles. All the primers used for qPCR are listed in Supplementary Table S2.

### Determination of contents of OPDA, JA, and JA-Ile

The contents of jasmonic acid (JA), JA-conjugated with isoleucine (JA-Ile), and the JA precursor 12-oxo phytenoic acid (OPDA) were quantified using ultra-performance liquid chromatography coupled with tandem mass spectrometry (UPLC-MS/MS) according to the method described by Balcke *et al.* (2012). Samples of ~50 mg of frozen middle-aged leaves (according to van Buer *et al.*, 2019) were homogenized in 500 μl methanol supplemented with 50 ng [^2^ H_5_]-OPDA, 50 ng [^2^ H_6_]-JA, and 50 ng [^2^ H_2_]-JA-Ile as internal standards. After sedimentation of insoluble plant material, the supernatant was diluted with nine volumes of water, passed through HR-XC columns (Macherey-Nagel) for solid-phase extraction, and eluted with 900 μl acetonitrile. Then, 10 μl of the eluate was subjected to UPLC-MS/MS. The OPDA, JA and JA-Ile contents were calculated based on the signals of the internal standard and the exact fresh weight used for extraction of each sample.

### Induction of estradiol-regulated expression in the *tAPX*-iOE and the *tAPX*-iRNAi lines

Transgene expression was induced in the *tAPX*-iOE lines without any cold treatment and in the *tAPX*-iRNAi lines immediately after cold-priming by spraying the plants at 20 °C from the top with 100 μM estradiol (Carl Roth GmbH + Co. KG, Karlsruhe, Germany) dissolved in 0.8% (v/v) DMSO and supplemented with 0.01% (v/v) Tween, as described by van Buer *et al.* (2019). Control plants were sprayed with a mock solution containing DMSO and Tween only.

### OPDA and JA spraying treatments

Plants were sprayed with 100 µM (3RS, 7RS)-(+/–)-–jasmonic acid (J0936; Duchefa, Haarlem, Netherlands) or 10 µM OPDA (8-{(1S,5S)-4-Oxo-5-[(2Z)-2-penten-1-yl]-2-cyclopenten-1-yl}octanoic acid; Cayman chemicals, Ann Arbor, USA) in 0.1% (v/v) ethanol 2 h before triggering and leaves were sampled after 1 h of triggering. Control plants were mock-treated with ethanol only.

### Statistical analysis

Construction of box-plots and multiple comparisons using ANOVA followed by Tukey–Kramer *post hoc* tests were performed in R v.4.0.4. Pearson correlation coefficients were calculated and depicted using PAST3 v.1.0 (Harper and Ryan, 2001). Student’s *t*-test was used for pairwise comparisons.

## Results

### Generation, selection, and evaluation of RNA-seq data

To screen Arabidopsis for priming-dependent regulation, 4-week-old plants were exposed for 24 h to 4 °C, returned to 20 °C for 5 d, and then subjected to triggering either by exposure to cold (4 °C) or high light (800 µmol m^–2^ s^–1^; Fig. 1). The changes in the transcriptome were examined from 0–120 min during exposure to the triggering conditions. Pair-end sequencing of cDNA libraries yielded 56.2–85.2 million reads per sample (Supplementary Table S3), of which 96.1–96.6% could be aligned to 27 860 transcript identities in the TAIR10 reference genome.

If the expression intensity for a large number of genes suddenly changes, as in the response to sudden exposure to cold or high light (Hahn *et al.*, 2013; Crisp *et al.*, 2017), it can cause a bias in the transcriptome. We found that when the FPKM values for eight widely constitutively expressed genes (*At1g13320*, *At1g07920*, *At2g28390*, *At2g29960*, *At4g27960*, *At4g33380*, *At5g46630*, and *At5g08290*; Weber *et al.*, 2004; Han *et al.*, 2013) were normalised to the C0 value across all the individual treatments, the overall mean was 1.02±0.05 (±SD) (Supplementary Table S4). Being very close to one, this value excludes the possibility of there being technical bias in the results. As an additional control, we examined the transcript levels of 20 genes that have previously been defined as ‘first-wave’ cold-responsive genes (Park *et al.*, 2015) and that were represented with an FPKM of at least 0.5 in C0 plants, and found that considerable accumulation had occurred following the first 30 min of cold exposure (C30C/C0; Supplementary Fig. S2). Across the whole the transcriptome, 88% of the genes previously described as being cold-induced after 3 h at 0 °C (Lee *et al.*, 2005) were up-regulated by at least 1.5-fold following the first hour at 4 °C (C60C/C0; Supplementary Table S5A). After 60 min in heat-filtered, high light, more than two-thirds of genes that have previously been identified as being regulated by high light (Crisp *et al.*, 2017; but note it was not heat-filtered) were up- or down-regulated by at least 1.5-fold (C60L/C0; Supplementary Table S5B). We performed qPCR after reverse-transcription of mRNA in the RNA samples from three independently cultivated and treated sets of plants and the results confirmed the reliability of the RNA-seq data (Supplementary Fig. S3).

**Fig. 2.**
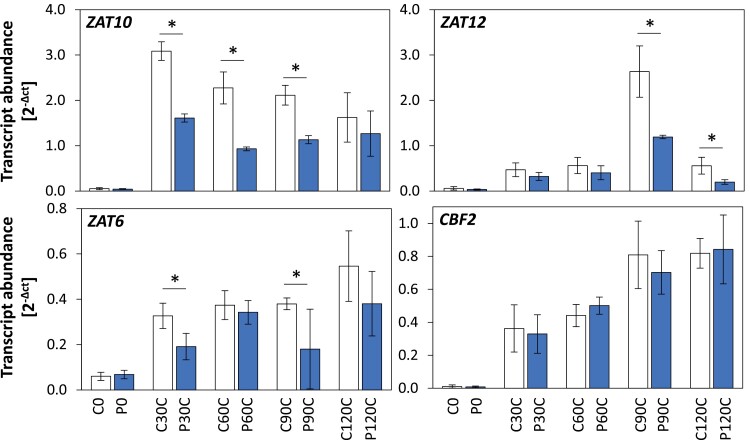
Priming-dependent expression of *ZAT10*, *ZAT12*, *ZAT6*, and *CBF2* in Arabidopsis in response to cold-triggering. Plants were subjected to cold-priming at 4 °C for 24 h followed by 5 d under normal growth conditions at 20 °C, after which they were subjected to cold-triggering at 4 °C for 0–120 min. See Fig. 1 for treatments and abbreviations. Transcript abundances after 0, 30, 60, 90, and 120 min in control (C) and primed (P) plants were determined using qPCR and normalized on the geometric mean of the abundances of *YLS8* and *CYP5*. Significant differences between the control and cold-primed plants were determined using Student’s *t*-test: *P<0.05 (*n*=3–4).

Our RNA-seq time-course experiment served us as a tool to filter the transcriptome for priming regulation. False-positive effects were minimized by setting the threshold to 1.5-fold change (higher or lower than the relevant control level) for defining genes as being priming-regulated during triggering. This value corresponds to twice the maximum amplitude observed using qPCR for the most variable of the genes, that were suggested by Han *et al.* (2013) as constitutively expressed (Supplementary Table S4). This threshold provides a very strong and highly stringent filter, to the extent that even some previously identified primable genes did not pass it, for example *ZAT10*, *ZAT6*, and *ZAT12* (van Buer *et al.*, 2016, 2019; Bittner *et al.*, 2020) (Supplementary Table S4), although qPCR analysis showed that they were primed (Fig. 2). Transcript levels of *CBF2* (*At4g25470*) increased gradually (similar to the pattern observed for *ZAT6*), but no priming effect was observed during the 2-h course of the experiment.

We applied high thresholds in this study because the reliability of scoring genes as being regulated by priming was more important than determination of the precise number of primable genes. In addition, we excluded weakly expressed and weakly regulated genes by setting the minimum FPKM threshold in C0 and P0 to 0.5, the threshold for the mean FPKM over all 14 samples to 2, and the threshold for stressor-specific regulation to 1.5-fold after 30, 60, and 120 min of cold- or light-triggering (C30/C0; C60/C0, and C120/C0). Finally, we removed all data for 36 miscellaneous RNAs, 147 RNAs representing uncharacterized or hypothetical proteins, and 122 short non-coding RNAs as they would not give any information on functional gene categories in the final step of the analysis and would have required higher quality technical data for reliable analysis. A total of 1860 genes with priming-dependent regulation of expression passed all these pre-selection criteria (Supplementary Table S6).

### Analysis of the priming effect on triggering responses

**Fig. 3.**
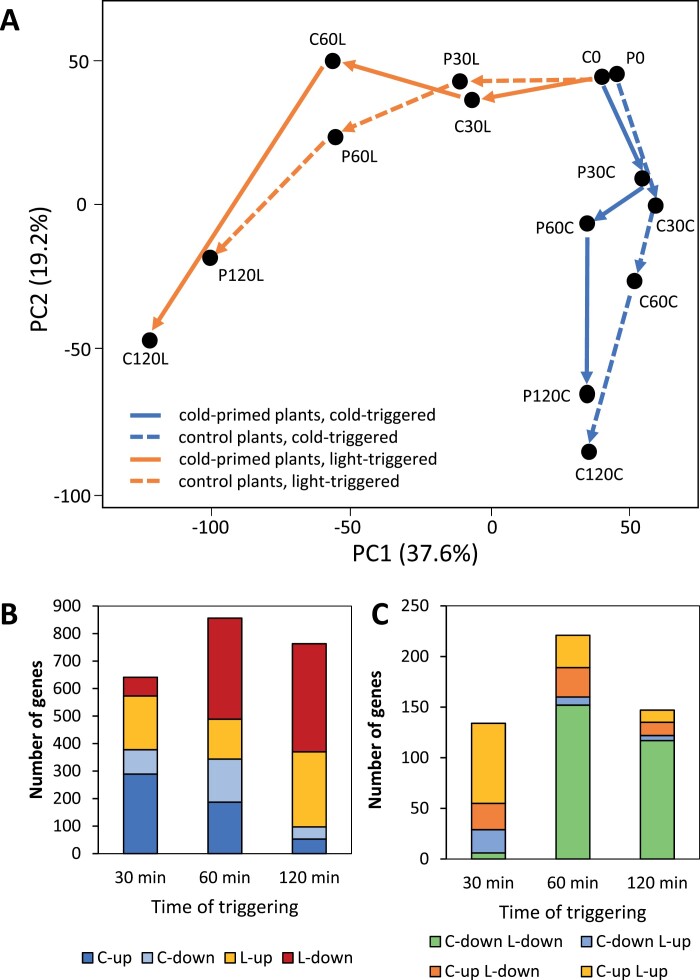
Genome-wide analysis of transcript variance and number of genes regulated by cold-priming during cold- and light-triggering in Arabidopsis. Plants were subjected to cold-priming at 4 °C for 24 h followed by 5 d under normal growth conditions at 20 °C, after which they were subjected to cold-triggering at 4 °C for 0–120 min. See Fig. 1 for treatments and abbreviations. (A) Principal component (PC) analysis of transcript levels based on single-value decomposition (SVD) of cold-primed (P) and control (C) plants during 2 h of cold- and light-triggering. (B) Numbers of genes significantly up- and down-regulated after 30–120 min of triggering by either light (L) or cold (C) in a priming-dependent manner (Primed>1.5×Control or Primed<1/1.5×Control) according to the RNA-seq data set, and (C) numbers of genes significantly up- and down-regulated after 30–120 min of triggering by light and cold in a priming-dependent manner.

The transcriptomes of C0 and P0 plants obtained 5 d after priming and immediately before triggering were very similar (Fig. 3A), which was consistent with our previous study (Bittner *et al.*, 2020) and demonstrated that the plants generally recovered from priming-induced regulation of transcript abundance. Principal component analysis across all 14 samples identified the triggering stresses as the strongest drivers for separating the RNA-seq profiles (37.6% of the variance, PC1; Fig. 3A). The duration of stress exposure was the second-highest variance component (19.2%, PC2). The P and C samples differed most after 60 min (Fig. 3). Many transcript levels were up-regulated in a priming-dependent manner after 30 min, especially in the light, whilst there was dampening of the response of many genes between 30 min and 60 min, both in the light and in the cold (Fig. 3B). For many of these genes, the priming-effect was already manifested after 30 min in the cold. The positive and negative priming effects on cold-triggering decreased between 60 min and 120 min, whilst the light-specific priming effects diversified. After 2 h, the transcriptomes showed the trigger-specific characteristics known from our previous analysis (Bittner *et al.*, 2020).

To uncouple regulation during triggering from the absolute expression levels, the FPKM values of all genes were normalized to their C0 values. For functional characterization, the data were classified according to the trigger-specificity of the priming response and clustered according to the gene expression profiles. Of the priming-sensitive genes that showed a response in the FPKM value of more than 1.5-fold in the P30/C30, P60/C60, and P120/C120 comparisons, 374 out of 1860 genes (20%) responded only to cold-triggering, 900 (48%) only to high light-triggering, and 586 (32%) to both cold- and light-triggering (Supplementary Table S6).

#### Genes showing priming effects during both cold- and light-triggering

**Fig. 4.**
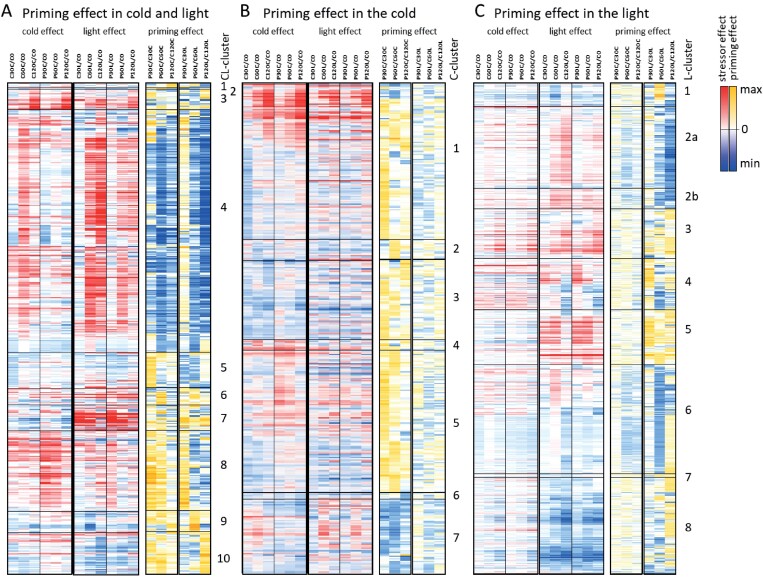
Heatmaps depicting stressor-dependent and priming-dependent regulation of gene expression in Arabidopsis during 2 h of cold- and light-triggering. Plants were subjected to cold-priming at 4 °C for 24 h followed by 5 d under normal growth conditions at 20 °C, after which they were subjected to cold-triggering at 4 °C for 0–120 min. See Fig. 1 for treatments and abbreviations. All heatmaps are zero-centred. (A) Heatmaps of triggering-induced regulation of 586 genes that were identified as being regulated by cold-priming in both cold- and light-triggering conditions (the CL cluster). The genes are arranged in the order of Supplementary Table S6 according to the profile-based cluster analysis shown in Supplementary Fig. S4A. For the heatmap showing regulation during triggering, the FPKM values of all genes were normalized to that in the control plants immediately prior to triggering (C0). For the heatmap showing priming-dependent regulation, the FPKM-values in primed plants at each time-point were normalized to the corresponding values of the corresponding control plants. (B) Heatmaps of triggering-induced regulation of 374 genes that were identified as being regulated by cold-priming only in the cold-triggering conditions (the C cluster). The genes are arranged in the order of Supplementary Table S6 according to the profile-based cluster analysis performed with ClustVis and depicted in Supplementary Fig. S5A. The data were normalized as described in (A). (C) Heatmaps of triggering-induced regulation of 900 genes that were identified as being regulated by cold-priming only in the light-triggering conditions (the L cluster). The data were normalized as described in (A). The genes are arranged in the order of Supplementary Table S6 according to the profile-based cluster analysis depicted in Supplementary Fig. S6A.

These genes formed 10 clusters (referred to as CL-clusters; Fig. 4A, Supplementary Fig. S4A). The largest one of them, CL-cluster 4, grouped 290 cold- and light-inducible genes that were dampened in their response 5 d after cold-priming. A total of 197 of these genes were JA-inducible and the vast majority of them respond quickly to JA (within 60 min; Supplementary Tables S6A, S7; Hickman *et al.*, 2017). This group included a full set of genes required for the oxylipin pathway leading to JA-biosynthesis, namely lipoxygenases (*LOX2–4*; *At3g45140*, *At1g17420*, and *At1g72520*), allene oxide synthase (*AOS*; *At5g42650*), allene oxide cyclases (*AOC1–3*; *At3g25760*, *At3g25770*, and *At3g25780*), OPC-8:0 CoA ligase1 (*OPCL1*; *At1G20510*), and oxophytodienoate-reductase (*OPR3*; *At2g06050*), or JA side-reactions (hydroperoxide lyase 1, *HPL1*; *At4g15440*)), and key elements of JA-signalling, such as JAZ proteins and WRKY transcriptions factors (Supplementary Table S6A, Table 1) (Feussner and Wasternack, 2002).

**Table 1.**
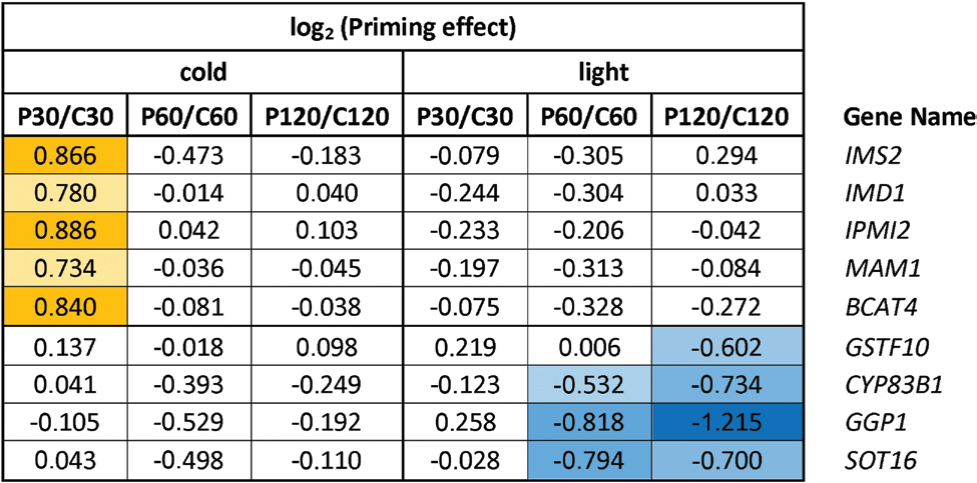
Priming effects on the expression of glucosinolate biosynthesis genes during cold- and light-triggering

Data are the ratios of expression in the primed (P) plants relative to the control (C) plants at the different sampling time-points, as shown in Fig. 1. The data were extracted from Supplementary Table S6 and log_2_-transformed. Positive priming effects are indicated in yellow and negative effects are indicated in blue, with darker shading indicating stronger effects in each case.

Many genes in CL-clusters 7 and 8 were inversely priming-regulated in the cold and in the light (Fig. 4A), for example *PCC1* (*At1g21250*) and *BAP1* (BON-associated protein 1; *At3g61190*) (Supplementary Table. S6A), for which inverse regulation has previously been demonstrated using qPCR after prolonged exposure to cold and high light (Bittner *et al.*, 2020). Many of the genes in the two clusters were associated with functions related to biotic stress, defence, and systemic acquired resistance Supplementary Fig. S4B). In contrast to the CL-cluster 4 genes, those of clusters 7 and 8 were identified as being preferentially negatively JA-regulated (Hickman *et al.*, 2017), and 12 of them are also known to be sensitive to the JA-signalling modulator salicylic acid (SA; Blanco *et al.*, 2009; Supplementary Table S8), a feature not shared with any of the genes in cluster 4. The other CL-clusters were smaller and showed only weak indications of GO enrichments, for example drought-related functions (cluster 6), gibberellic acid and processes related to lipid transport (cluster 9), and wax and polysaccharide biosynthesis (cluster 10) (Supplementary Fig. S4C, D).

#### Cold-priming effects on cold-triggering

The 374 genes that were preferentially priming-regulated during cold-triggering formed seven clusters (referred to as C-clusters; Fig. 4B, Supplementary Table S6B, Supplementary Fig. S5A). As in the CL-clusters, positive priming effects (P>1.5×C) widely correlated with down-regulation of transcript levels (C30, C60, C120 <C0) in the cold in non-primed plants and negative effects (P<1/1.5×C) with cold-induction in non-primed plants (C30, C60, C120 >C0) (Fig. 4B). Dampening of the cold-response was especially strong at the top of C-clusters 1 and 7 after 30 (–60) min of triggering (Fig. 4B, Supplementary Fig. S5B). The two clusters had slight JA imprints and shared similarity in expression with CL-cluster 4, except that the light response was not as strong (Supplementary Table S7). C-Cluster 1 contained *ORA59* (*At1g06160*), which together with the C-cluster 7 gene *ERF1* (*At1g28370*) mediates MYC2-controlled ethylene signalling (Çevik *et al.*, 2012), and grouped together also several genes for enzymes of the isopropylmalate dehydrogenase (IPMDH) branch of glucosinolate biosynthesis, namely *IMS2* (*At5g23020*), *IMD1* (*At5g14200*), *IPMI2* (*At2g43100*), *MAM1* (*At5g23010*), and *BCAT4* (At3g19710) (Harun *et al.*, 2020; Supplementary Fig. S5B). C-Cluster 3, the transcript levels of which were stable for longer in the cold, grouped many genes for stress- and hormone-responsive transcriptions factors together with ones regulating development, such as *MYB26* (*At3g13890*), *MYB28* (*At5g61420*), *MYB88* (*At2g02820*), and *bHLH68* (*At4g29100*) (Supplementary Fig. S5B, Supplementary Table S6B). C-Cluster 5 was characterized by genes that had rapid accumulation of transcripts in the primed plants in response to cold-triggering but down-regulation in the non-primed ones (Fig. 4B). Several of these genes, in common with the rapidly activated genes of C-cluster 4, are associated with functions in immune and stress responses (Supplementary Fig. S5C), which is consistent with our recent studies on the effects of cold-priming on the responses to subsequent cold, light, and pathogen treatments (Bittner *et al.*, 2020; Griebel *et al.*, 2020, Preprint).

#### Cold-priming effects on light-triggering

The 900 genes with stronger priming effects in high light formed eight clusters (referred to as L-clusters; Supplementary Table S6C, Supplementary Fig. S6A). Of these genes, 307 were JA-sensitive (Supplementary Table S7). Similar to the majority of the CL- and C-cluster genes, the genes in L-clusters 1, 2, 6, 7, and 8 were dampened in their light response in primed plants (Fig. 4C). Strong dampening of the responses in L-clusters 2 and 6 correlated with over-representation of positively JA-regulated genes (114 of 158). Similar to CL-cluster 4. L-cluster 2 grouped together genes supporting biosynthesis of defence and signalling molecules (Supplementary Table S7), such as aromatic amino acids (Supplementary Fig. S6B) and indolyl glucosinolates (Ludwig-Müller *et al.*, 2000; Sasaki-Sekimoto *et al.*, 2005), whilst L-cluster 6 contained genes encoding enzymes involved in the biosynthesis of long fatty acids (e.g. *At3g48720* and *At2g28630*), TIR-NBS and CC-NBS immune receptors (e.g. *At5g45060* and *At2g59620*), and the heat-shock transcription factor *HSFC1* (*At3g24520*) (Supplementary Table S8). The transcript levels of these genes were considerably decreased transiently around 60 min in primed plants in the light. Regulation of the genes in the bottom part of L-cluster 6 (Fig. 4C) up to 60 min of cold triggering was similar to the regulation during light triggering, except that it was weaker. However, in the light only, these genes were down-regulated between 60 min and 120 min.

In contrast, priming increased the transcript abundances in L-cluster 3, and this was even more pronounced in L-clusters 4 and 5 (Fig. 4C). Several genes associated with cold and drought acclimation, such as *CBF1* (*At4g25490*), *DREB1A /CBF3* (*At4g25480*), fatty acid reductase 1 (*FAR1*; *At5g22500*), and delta-9-desaturase 1 (*ADS1*; *At1g06080*) (Supplementary Table S6C), belong to L-cluster 3 and genes related to osmotic stress and protein repair (Supplementary Fig. S6D), such as *HSP*s (*At5g53400*, *At5g52640*, *At2g32120*, and *At1g74310*) to L-cluster 5 (Supplementary Table S6C). L-cluster 4 was characterized by genes that quickly responded to excess light, for example, those for the transcription factors *ERF5* (*At5g47230*), *DREB2A* (*At5g05410*), and *WRKY6* (*At1g62300*) (Fig. 4C, Supplementary Table S6C). This cluster also contained the genes for the zinc-finger transcription factors *ZAT6, ZAT10,* and ZAT12. These clustered closely together and showed positive priming effects after 30 min of light-triggering, but no or only slightly negative effects upon longer exposure (Supplementary Table S6C), consistent with the regulation previously described after prolonged light-triggering of cold-primed plants (Bittner *et al.*, 2020).

### The early response to cold and light stress in primed plants shows a distinct JA signature

In our study of early priming effects, about a third of the genes that showed priming-dependent regulation during the first 2 h were associated with JA sensitivity (Supplementary Table S7). This is a higher proportion than in previous priming studies on regulation of systemic acquired resistance (Pozo *et al.*, 2008) and dehydration responses (Ding *et al.*, 2013). In parallel, we found much weaker responses for abscisic acid (ABA) and SA in our data than in the dehydration and resistance studies (Supplementary Table S6). For example, the transcript levels of the ABA-sensitive gene *RD29*, which is the reference gene for the [+/+]-cluster of dehydration induced dehydration memory-regulated genes that are stronger induced by dehydration-triggering after dehydration priming (Liu and Avramova, 2016), were only slightly positively regulated by cold-priming during light-triggering and down-regulated by ~10 % during cold-triggering, even though cold-signalling shares high similarities with drought-/dehydration-signalling (Shinozaki and Yamaguchi-Shinozaki, 1996). Further differentiating cold-priming from dehydration priming, the transcript levels of *PR2* (*At3g57260*) and *PR5* (*At1g75040*), which report high SA levels (Srinivasan *et al.*, 2009), were not increased in P0 plants (Supplementary Tables S6, S8) and expression of negatively JA-sensitive genes was increased in the cold and in the light (e.g. CL-cluster 8).

With 48% of its genes being related to JA, CL-cluster 4 showed the strongest JA-signature Table 2. In addition, it gave no indication for SA interference (Supplementary Tables S7, S8). Of the 290 genes in this cluster, 154 overlapped with a previously described cluster of dehydration-priming-responsive, non-epigenetically controlled [+/–]-genes (Ding *et al.*, 2013; Table 2), which are, like the CL-cluster 4 genes, induced by the stressor in non-primed plants, but show less or no induction by the same stressor in primed plants (Ding *et al.*, 2012). Subsequent analysis of the [+/–]-cluster excluded epigenetic regulation of the dehydration-priming-sensitive genes (Avramova, 2019). Consistently, subdivision of the CL-cluster 4 into two groups with either putative sensitivity or insensitivity to histone-3 trimethylation (according to Vyse *et al.*, 2020; Supplementary Table S6) also distinguished the cold priming effect from epigenetic regulation (Supplementary Fig. S4B).

**Table 2.** Numbers of genes overlapping between those identified by Ding *et al.* (2013) as being regulated by dehydration-priming and clusters of genes identified in the current study as showing cold-priming effects in response to cold-triggering only, light-triggering only, and both cold- and light-triggering

	Cluster and number of genes					
Ding *et al.* category and no. of genes	CL-cluster (586)	C-cluster (374)	L-cluster (900)	CL-cluster 4 (290)	CL-cluster 7 (31)	CL-cluster 8 (96)
[+/+], 362	31	12	34	10	4	5
[+/–], 857	**172**	38	139	**154**	3	5
[–/+], 434	19	12	47	1	2	7
[–/–], 310	12	10	8	4	0	7

Ding *et al.* categories are as follows: [+/+], memory genes were induced in response to dehydration stimulus and induced to higher levels in primed plants; [+/–], memory genes were induced in response to dehydration and were less induced in primed plants; [–/+], memory genes were down-regulated in response to dehydration stimulus and less regulated in primed plants; [–/–], memory genes were down-regulated in response to the dehydration stimulus and regulated to lower levels in primed plants. Current study clusters: CL, cold-priming effects in response to both cold- and light-triggering; C, cold-priming effects in response to cold-triggering only; L, cold-priming effects in response to light-triggering only. Data are also shown for individual subclusters within the CL cluster (Supplementary Table S6). Significant overlaps of the Ding *et al.* results with the CL-cluster and CL-subcluster 4 are highlighted in bold.

### qPCR analysis of CL-cluster 4 genes for priming-regulation

**Fig. 5.**
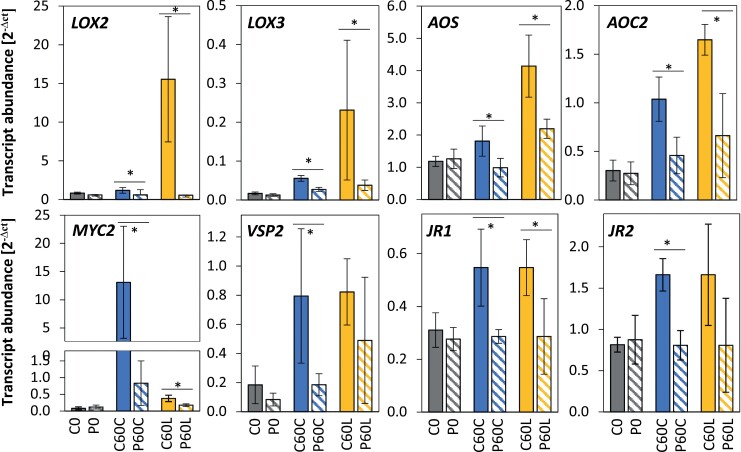
Effects of cold-priming on expression of genes responsive to jasmonic acid (JA) in Arabidopsis in response to cold- and light-triggering. Plants were subjected to cold-priming at 4 °C for 24 h followed by 5 d under normal growth conditions at 20 °C, after which they were subjected to cold-triggering at 4 °C for 0–120 min. See Fig. 1 for treatments and abbreviations. *LOX2*, *LOX3*, *AOS* and *AOC2* are involved in JA biosynthesis, and *MYC2*, *VSP2*, *JR1*, and *JR2* are other genes that are responsive to JA. Transcript abundances after 0 min and 120 min in control (C) and primed (P) plants were determined using qPCR and normalized on the geometric mean of the abundances of *YLS8* and *CYP5*. Data are means (±SD) of *n*=4–5 independent biological replicates. Significant differences between means were determined using Student’s *t*-test: *P<0.05.

The strong, co-dampening effect on the response of several genes for early enzymes in the biosynthesis of JA and other oxylipins after cold and dehydration priming suggests that they are core targets and multipliers of priming-regulation (Table 3). To begin our examination of the mechanisms behind the strong JA imprint, we evaluated the regulation of transcript abundance of genes in the CL-cluster 4 for the JA biosynthetic enzymes LOX2, LOX3, AOC2, and AOS, together with the JA-sensitive genes *MYC2*, *JR1*, *JR2*, and *VSP2* after 60 min in either cold or high light. qPCR analysis of 3–4 independently grown and treated biological replicates confirmed that the genes are regulated by priming-dependent dampening (Fig. 5). The results also showed that the transcriptional master regulator of JA signalling, *MYC2* (Kazan and Manners, 2013), was more strongly induced and much more priming-sensitive in response to cold-triggering than light-triggering. In contrast, the genes for oxylipin biosynthetic enzymes were more strongly regulated in the light than in the cold. As *MYC2* was hardly expressed prior to triggering (C0 and P0), we concluded that priming-regulation of the oxylipin biosynthetic genes was not directly or solely controlled by the priming-regulation of *MYC2*.

**Table 3.**
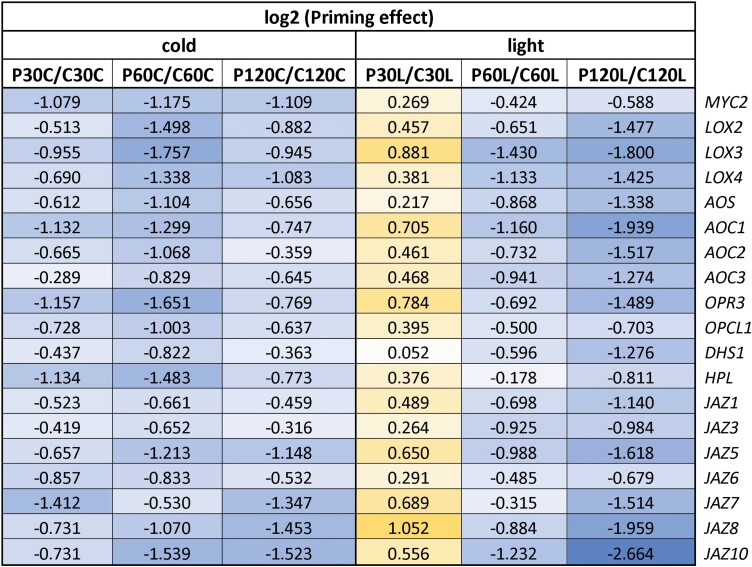
Priming effects on the expression of JA biosynthesis genes during cold- and light-triggering

Data are the ratios of expression in the primed (P) plants relative to the control (C) plants at the different sampling time-points, as shown in Fig. 1. The data were extracted from Supplementary Table S6 and log_2_-transformed. Positive priming effects are indicated in yellow and negative effects are indicated in blue, with darker shading indicating stronger effects in each case.

### Effects of *tAPX* expression on early priming-regulation and oxylipin biosynthetic genes

**Fig. 6.**
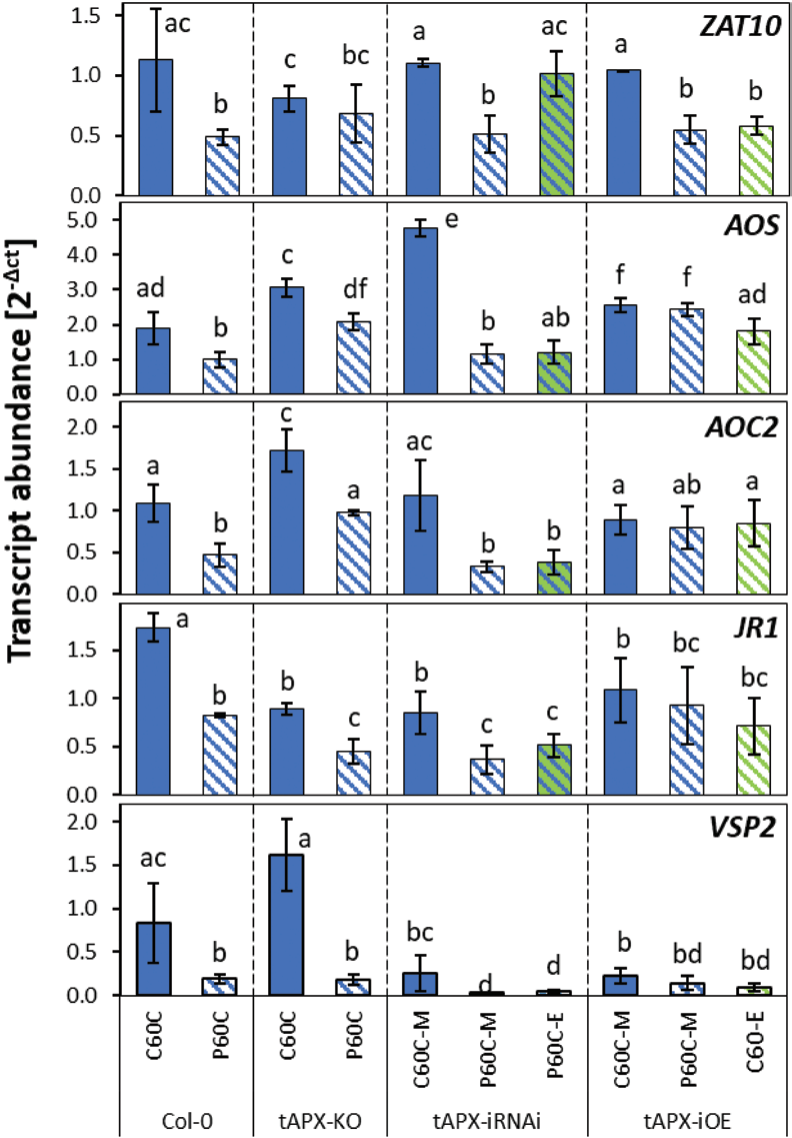
Effects of cold-priming on the expression of *ZAT10* (previously characterized for its tAPX-dependent priming regulation) and four JA-responsive genes of the CL-cluster 4 in different *tAPX* genetic backgrounds in Arabidopsis in response to cold-triggering. The Col-0 wild-type was used together with a well-established *tAPX*-knockout T-DNA insertion line (*tAPX-KO*), an estradiol-inducible *tAPX*-iRNAi line, and an estradiol-inducible t*APX*-overexpression (-iOE) line. Plants were subjected to cold-priming at 4 °C for 24 h followed by 5 d under normal growth conditions at 20 °C, after which they were subjected to cold-triggering at 4 °C for 60 min. Immediately after cold-priming, plants of the *tAPX*-iRNAi line were sprayed either with a solution containing estradiol (E) or a mock solution (M). The tAPX-iOE line was sprayed with the same solutions without any cold pretreatment. Transcript abundances in control (C) and primed (P) plants were determined using qPCR and normalized on the geometric mean of the abundances of *YLS8* and *CYP5*. Data are means (±SD) of *n*=3–5 independent biological replicates. Different letters indicate significant differences between means as determined using ANOVA followed by Tukey–Kramer *post hoc* tests (*P*<0.05).

Cold-priming regulation of the oxylipin-sensitive gene *ZAT10* (Taki *et al.*, 2005; Hickman *et al.*, 2017) is under the control of post-cold activation of *tAPX* expression (van Buer *et al.*, 2019). Given that tAPX is a major enzyme that protects plants from photooxidative stress, such as that caused by sudden decreases in temperature or increases in light intensity (Huner *et al.*, 1993; Fryer *et al.*, 2002; Maruta *et al.*, 2012) and supporting lipid peroxidation (Robert-Seilaniantz *et al.*, 2011; Zander *et al.*, 2020), we examined the CL-cluster 4 genes for *tAPX*-dependent regulation by qPCR in three independently cultivated sets of plants that had been cold-primed for 24 h, returned to normal conditions for 5 d, and then cold-triggered for 60 min. In the *tAPX*-knockout (-KO) line, *AOS*, *AOC2*, *JR1*, and *VSP2* were, unlike *ZAT10*, cold-primable (Fig. 6). All these genes, but not *ZAT10*, remained cold-primable in the estradiol-inducible *tAPX*-iRNAi line, in which the cold-priming-induced accumulation of tAPX was antagonized by the RNAi. Also different from *ZAT10* regulation, estradiol-induced transient overexpression of *tAPX* (*tAPX*-iOE) at 20 °C did not mimic the cold-priming-effect on *AOS*, *AOC2*, *JR1*, and *VSP2* (Fig. 6). The difference demonstrated that that the CL-cluster 4 genes were, unlike the previously characterized stress response mediating gene *ZAT10*, regulated via a tAPX-independent pathway.

### Cold-priming effects on the contents of JA, JA-Ile, and OPDA

**Fig. 7.**
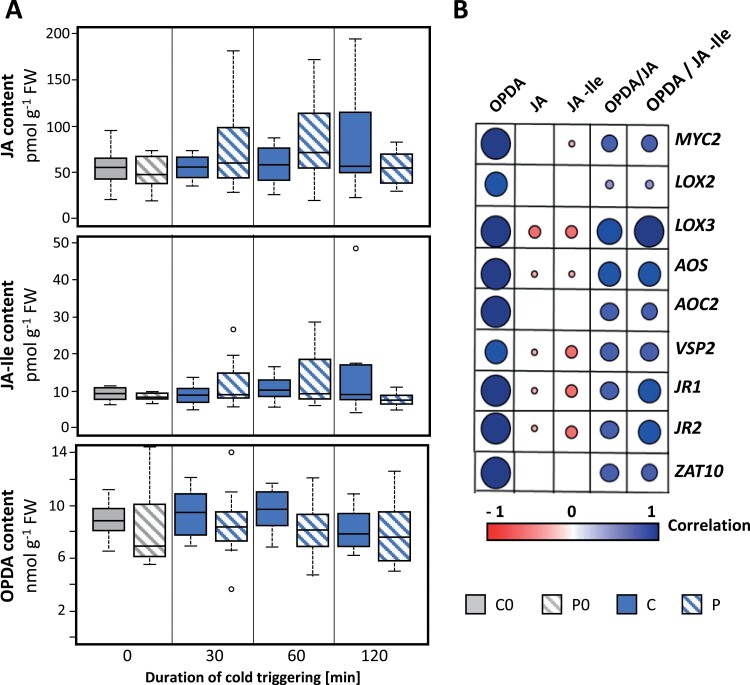
Effects of cold-priming on jasmonic acid (JA) metabolism in Arabidopsis in response to cold-triggering. Plants were subjected to cold-priming at 4 °C for 24 h followed by 5 d under normal growth conditions at 20 °C, after which they were subjected to cold-triggering at 4 °C for 0–120 min. (A) Endogenous concentrations of JA, JA-Ile, and the JA precursor 12-oxo phytenoic acid (OPDA) in middle-aged leaves as defined in van Buer *et al.* (2019). The boxplots show the median, the 25–75% interquartile range, and the distribution of the data (*n*=12). (B) Pearson correlations between concentrations of JA, JA-Ile, and OPDA, and the OPDA/JA and OPDA/JA-Ile ratios and expression levels of genes responsive to JA in primed plants after 60 min of cold-triggering.

Herbivore- and wounding-induced priming responses have been explained by increases in JA biosynthesis and dehydration-priming effects have been associated with decreases (Chung *et al.*, 2008; Chini *et al.*, 2009; Liu *et al.*, 2016). To test cold-priming effects on oxylipin biosynthesis, we used HPLC-MS-MS to determine the concentrations of OPDA, JA, and JA-Ile in middle-aged leaves, which are known to be the most priming-sensitive ones within the rosettes of 4-week-old plants (van Buer *et al.*, 2019). The JA and JA-Ile concentrations were very low (pmol range) and close to the detection level (Fig. 7A). After 30 min and 60 min of cold-triggering, the concentrations tended to be slightly higher in primed plants than in non-primed ones. In contrast, the concentration of OPDA tended to be lower in the primed plants at these time-points. Pairwise comparisons of the regulation of the concentrations of OPDA, JA, and JA-Ile, and the OPDA/JA and OPDA/JA-Ile ratios with regulation of transcript abundance level showed strongest correlations with OPDA (Fig. 7B). In parallel, only weak, negative correlations were found for JA and JA-Ile concentrations for *LOX3*, *AOS*, *VSP2*, *JR1*, and *JR2*.

### Effects of JA and OPDA treatment on priming

**Fig. 8.**
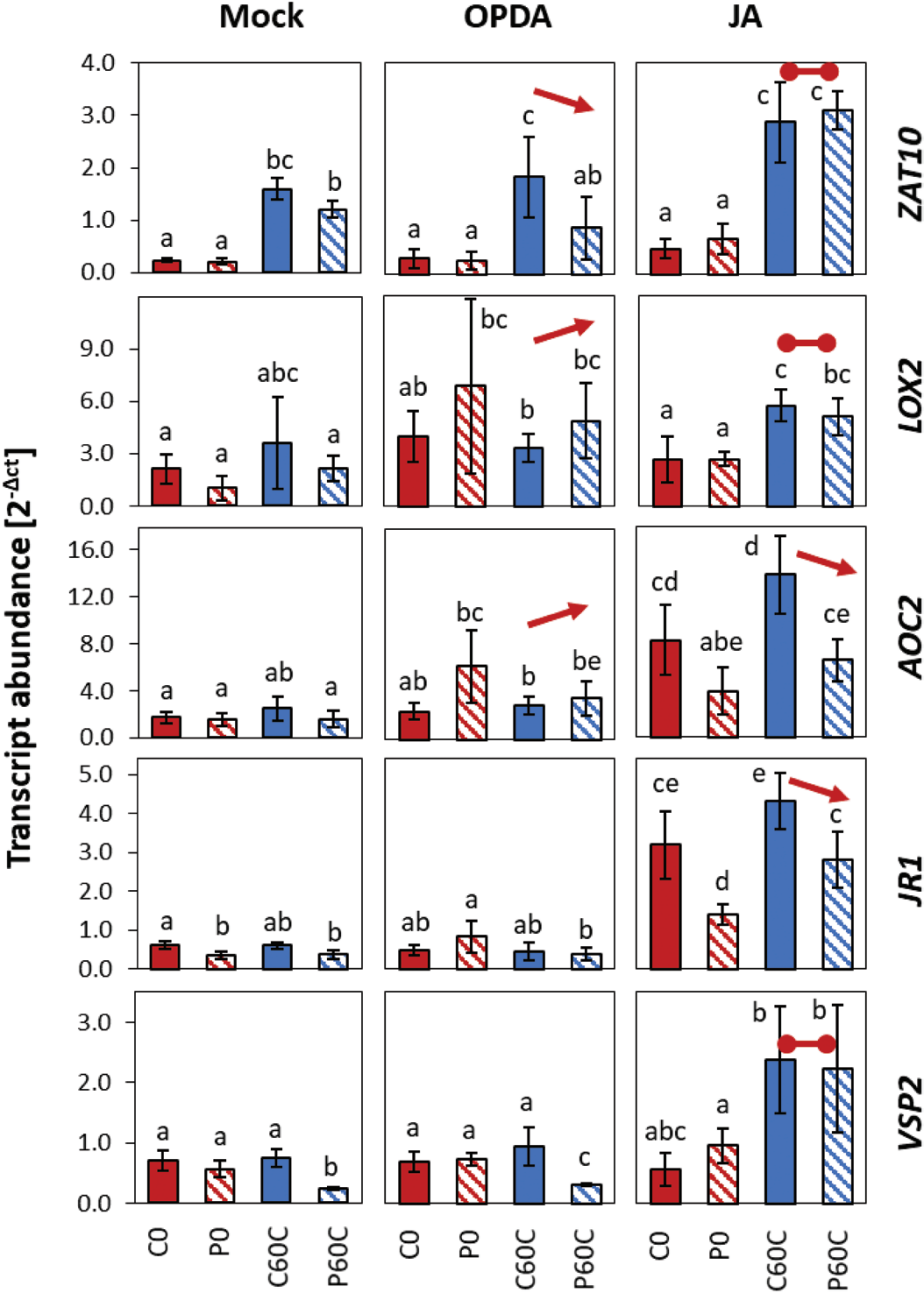
Effects of 12-oxo phytenoic acid (OPDA) and jasmonic acid (JA) on priming-dependent regulation in Arabidopsis of four JA-related CL-cluster 4 genes and *ZAT10* in response to cold-triggering. Plants were subjected to cold-priming at 4 °C for 24 h followed by 5 d under normal growth conditions at 20 °C, after which they were subjected to cold-triggering at 4 °C for 60 min. At 2 h before triggering, the plants were sprayed with either 10 µM OPDA, 100 µM JA, or a mock solution, and entire rosettes of plants were sampled after 60 min of triggering. Transcript abundances in control (C) and primed (P) plants were determined using qPCR and normalized on the geometric mean of the abundances of *YLS8* and *CYP5*. The four JA-related genes are all in CL-cluster 4 of genes showing priming effects during both cold- and light-triggering (Supplementary Table S6). Data are means (±SD) of *n*=3 independent biological replicates. Different letters indicate significant differences between means as determined using ANOVA followed by Tukey–Kramer *post hoc* tests (*P*<0.05). The arrows indicate significant increased or decreased regulation in cold-triggered plants compared to the mock control (i.e. comparison of P60C/C60C values). The horizontal lines indicate the absence of regulation in cold-triggered plants.

We next examined the effects of JA and OPDA on the expression of *AOC2*, *LOX2*, *JR1*, and *VSP2* and compared it with the effect of the independently cold-priming regulated, but also JA-sensitive gene *ZAT10.* We sprayed 4-week-old primed and non-primed Col-0 plants with either JA or OPDA 2 h before cold-triggering and determined transcript levels after 60 min of triggering using qPCR. For *ZAT10*, cold-induction and the cold-priming effect were maintained in plants sprayed with OPDA, but this was not the case for the four CL-cluster 4 genes (Fig. 8). The transcript levels for *LOX2* and *AOC2* (and to a lesser extent that of *JR1*) tended to be higher in the P0 plants than in the C0 ones before triggering. This also tended to be the case after 60 min triggering at 4 °C, thus suggesting that priming increased the OPDA-sensitivity of these genes. Spraying with JA resulted in increased cold-induction of all the genes (Fig. 8). Priming-dependent dampening of the response was lost for *ZAT10*, *LOX2*, and *VSP2*. The expression of *AOC2* and *JR1* showed the dampening effects, not only after cold-triggering, but also at 20 °C in the JA-sprayed plants, demonstrating dominance of priming-regulation over temperature- and JA-regulation.

### Effects of JA and OPDA biosynthesis on priming

**Fig. 9.**
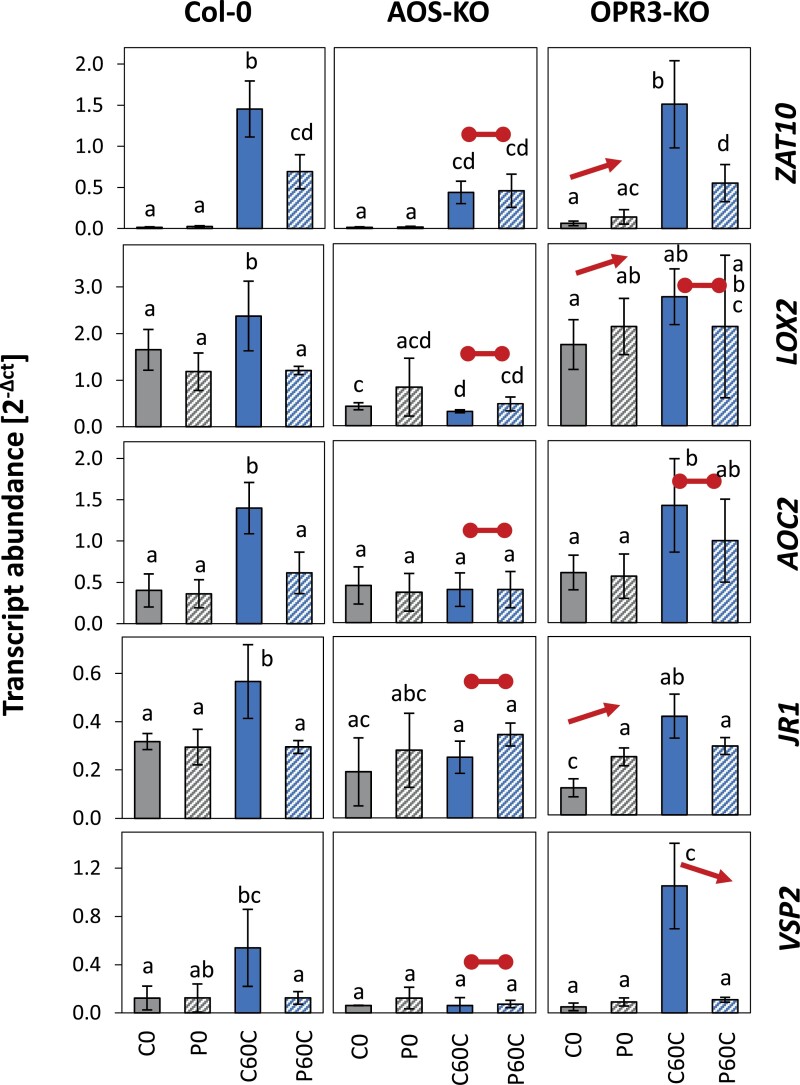
Effects of reduced synthesis of 12-oxo phytenoic acid (OPDA) and jasmonic acid (JA) on priming-dependent regulation in Arabidopsis of four JA-related genes and *ZAT10* in response to cold-triggering. The Col-0 wild-type was used together with an *AOS*-knockout (-KO) line, which lacks an early enzyme of the chloroplast part of the oxylipin pathway and has reduced levels of JA and OPDA, and an *OPR3*-KO line which is limited in the conversion of OPDA in to JA. Plants were subjected to cold-priming at 4 °C for 24 h followed by 5 d under normal growth conditions at 20 °C, after which they were subjected to cold-triggering at 4 °C for 60 min. Transcript abundances in control (C) and primed (P) plants were determined using qPCR and normalized on the geometric mean of the abundances of *YLS8* and *CYP5*. The four JA-related genes are all in CL-cluster 4 of genes showing priming effects during both cold- and light-triggering (Supplementary Table S6). ZAT10 served as a reference for the previously described tAPX-dependent cold-priming pathway. Data are means (±SD) of *n*=4 independent biological replicates. Different letters indicate significant differences between means as determined using ANOVA followed by Tukey–Kramer *post hoc* tests (*P*<0.05). The arrows indicate significant increased or decreased regulation in cold-triggered plants compared to the Col-0 wild-type (i.e. comparison of P0/C0 and P60C/C60C values). The horizontal lines indicate the absence of regulation in cold-triggered plants.

To investigate priming-regulation at low oxylipin levels, we compared priming-dependent regulation of *ZAT10*, *LOX2*, *AOC2*, *JR1*, and *VSP2* in the well-characterized *AOS*-KO line SALK_017756, which lacks allene oxid synthase and cannot synthesize ODPA and JA (Matschi *et al.*, 2015), and in the *OPR3*-KO line SALK_201355, which cannot convert OPDA to JA, because it is deficient in 12-oxophytodienoate reductase 3; Stintzi *et al.*, 2001; Wasternack and Hause, 2018) (Supplementary Fig. S1). In the absence of OPDA and JA, only *ZAT10* was cold-inducible. At this relatively low induction level, *ZAT10* did not show priming-dependent regulation (Fig. 9). In the *OPR3*-KO line, the availability of OPDA was sufficient to restore cold induction and the primability of *ZAT10* and *VSP2*, but not the primability of *LOX2* and *AOC2*, although these genes showed similar cold induction to the wild-type plants (Fig. 9).

## Discussion

### The first cold- and light-specific priming responses occur within the first 30 min of cold-triggering

By the time of our first measurements after 30 min of triggering, 389 genes had already shown stressor- and priming-specific (>1.5-fold) regulation in the cold and 207 in the light (Fig. 3B). At the three time-points only 16–21% of the priming regulated genes were priming-regulated both by cold- and by light-triggering (Fig. 3C), although cold and light regulate many genes in the same direction in non-primed plants (Bode *et al.*, 2016). These initial responses showed trigger-specific regulation of genes involved in the biosynthesis of aliphatic glucosinolate (IPMDH branch) (C-cluster 1) and indolic glucosinolate (L-cluster 2) (Supplementary Table S6) that support biosynthesis of precursors of bioactive and cytotoxic isothiocyanates (Andersson *et al.*, 2015; Harun *et al.*, 2020), of genes involved in regulators of the innate immune response (Clay *et al.*, 2009), and of genes involved in precursors and antagonists of auxin (Hull *et al.*, 2000; Katz *et al.*, 2015). Combined with early priming-regulation of many transcription factors such as MYBs, WRKYs, ERFs, and ZATs (Fig. 2, Supplementary Table S7), these genes stabilize divergent, trigger-specific, priming-dependent regulation, as previously described after prolonged triggering (Bittner *et al.*, 2020).

### Priming causes much stronger gene dampening than strengthening of regulation

Many priming studies have described increased activation of stress-induced genes upon triggering of primed plants (Ding *et al.*, 2013; Byun *et al.*, 2014; Conrath *et al.*, 2015; Crisp *et al.*, 2016; Zuther *et al.*, 2019). However, in our current study in which plants were subjected to a short cold stimulus followed by a 5-d lag period, only 10 genes were at least 1.5-fold stronger regulated in primed plants on top of at least 1.5-fold cold regulation. Eight positively priming-regulated genes (equivalent to only 0.43 % of all priming-responsive genes) were putative targets of cold-activated histone trimethylation (Supplementary Table S6), which regulates large groups of genes after priming with greater temperature shifts and/or triggering after shorter lag-phases (Velanis and Goodrich, 2017; Ganguly *et al.*, 2018; Friedrich *et al.*, 2019; Vyse *et al.*, 2020).

Several cold-inducible transcripts showed greater abundance in cold-primed plants when triggered by light, but fewer or none accumulated upon cold-triggering (L-clusters 3 and 5; Fig. 4). As both the cold- and light-triggered sets of plant were cold-primed in exactly the same way, these different responses demonstrate that positive priming imprints were actively overridden early during cold-triggering. Weaker regulation has been associated with cost-minimization and specification of transcriptional activity (Conrath *et al.*, 2015; Hilker *et al.*, 2016). As the dampening response strongly outweighed activation of stressor-specific genes in our study, we conclude that priming counteracts gene regulation when the plants experience cold for the second time after only a short pre-exposure and a long lag-phase. This could be important in spring, when short cold spells occur at low and irregular frequency whilst average temperatures are increasing. Priming-activated dampening then can protect plants from the activation of cost-intensive gene expression and metabolic shifts that produce greater cold-tolerance under persistent cold conditions, but which is too slow and not robust enough to establish protection after short spells of cold (Zuther *et al.*, 2015). Our study showed that at least two mechanisms were involved in the dampening response, one controlled by tAPX and the other tAPX-independent and associated with JA.

### Cold-priming causes a strong JA imprint by affecting the oxylipin sensitivity of genes encoding JA biosynthetic enzymes

A number of studies have reported JA imprints on the sets of genes that are differentially expressed as a result of priming by excess light, dehydration, cold, or pathogens (Gális *et al.*, 2009; Vos *et al.*, 2013; Liu *et al.*, 2016; Bittner *et al.*, 2020). Our analysis of the effects of priming on the responses to two different triggering stimuli identified a number of cold-priming-sensitive regulatory clusters (Fig. 4, Supplementary Table S6). The strongest effects occurred in two clusters within the genes that responded to both cold and light, namely CL-clusters 4 and 8.

CL-cluster 4 showed the strongest JA signature, with 48 % of the genes being JA-sensitive (and in contrast to CL-cluster 8, almost no genes were SA-sensitive) (Supplementary Tables S6–S8). This cluster grouped the full set of genes required for oxylipin biosynthesis together with many regulators of JA signalling, such as JAZ proteins (Feussner and Wasternack, 2002). Many genes were initially inversely regulated in the cold and in the light, but followed the initial cold response between 30 min and 60 min of triggering (Fig. 4, Supplementary Tables S7, S8), demonstrating that the effects of cold-priming overwhelmed the response to excess light. Overall, this cluster overlapped by 53% with a cluster of genes previously found to have dampened expression during dehydration stress following dehydration-priming (Ding *et al.*, 2013). The overlap covered the main genes required for the early steps of oxylipin biosynthesis, namely *MYC2*, *LOX2*, *LOX3*, *LOX4*, and *AOC1*Supplementary Table S6. The effect that we observed during light- and cold-triggering 5 d after 24-h cold-priming was much stronger than that observed by Ding *et al.* (2013) following triggering by dehydration 22 h after 2-h dehydration-priming (Fig. 5; Table 3; Supplementary Table S6). Our results also showed additional early co-priming regulation of *AOS*, *AOC2*, *AOC3*, *OPR3*, *OPCL1*, and *HPL*, which encode enzymes for further steps of the pathway, demonstrating that cold-priming affected JA biosynthesis at several levels. Most of these genes are themselves oxylipin-sensitive (Taki *et al.*, 2005; Hickman *et al.*, 2017), Hence, they can potentiate the primary effect and regulate secondary processes, such as growth, the defence-related glucosinolate pathway, and auxin metabolism (Supplementary Table S6, Table 1), as described in our previous study (Bittner *et al.*, 2020).

In a dehydration-priming study conducted by Avramova, (2017), dampening of JA-sensitive genes was explained by low *MYC2* expression at the end of a 22-h lag phase as a result of low (or no) availability of ABA. ABA biosynthesis and signalling, which activate or enhance *MYC2*-controlled JA signalling (Vos *et al.*, 2013; Liu and Avramova, 2016), are also induced in the cold and in response to light (Shinozaki and Yamaguchi-Shinozaki, 1996; Staneloni *et al.*, 2008). Comparison of the C0 and P0 transcriptomes showed that the primary ABA effects were removed during the long lag phase used in our study. Prior to cold- or light-triggering, *MYC2* transcript levels did not differ between the primed and control plants (Fig. 5). The JA concentrations were not decreased in cold-primed plants prior to cold-triggering and showed a trend towards accumulation in the first 60 min of the triggering process (Fig. 7). However, the transcript levels of all the CL-cluster 4 genes that we examined were lower in cold-primed plants after 60 min of cold-triggering (Fig. 5), demonstrating that attenuation of gene expression was activated in a priming-dependent manner during triggering. qPCR analysis in oxylipin biosynthetic mutants (Fig. 9) and after application of oxylipin to the plants (Fig. 8) showed that OPDA was required for induction of the genes in CL-cluster 4 and that priming was regulated by increased sensitivity of *LOX2* and *AOC2* to OPDA. By light-triggering, which stronger increased expression of the oxylipin-biosynthetic genes, dampening outweighed induction of the four examined oxylipin biosynthesis genes after 60 min (Fig. 5). By counteracting the stress-induced stimulation of JA biosynthesis, cold-priming gave the transcriptomes an exceptionally strong JA-signature, with about a third of the priming-sensitive genes being associated with JA-sensitivity.

### Priming of the oxylipin cluster genes is independent from regulation of ZAT10

Dampening of the oxylipin-sensitive genes was not controlled by tAPX, whereas regulation of the *MYC2* target *ZAT10* was (Fig. 6; van Buer *et al.*, 2019; Van Moerkercke *et al.*, 2019), which excludes the possibility of indirect effects, such as tAPX-mediated redox-modulation of oxylipin signalling (Satoh *et al.*, 2014) or feedback control of *ZAT10* on *MYC2* expression (Dombrecht *et al.*, 2007). As *ZAT10* remained slightly cold-inducible in the OPDA- and JA-deficient *AOS*-KO line, in which priming of *LOX2*, *AOC2*, *JR1* and *VSP2* was disturbed (Fig. 9), this indicated the bidirectional independence of CL-cluster 4 genes from regulation of ZAT10. Accumulation of OPDA in response to the triggering stimulus increased the responsiveness of the genes to JA in non-primed plants. In primed plants, the change in oxylipin sensitivity (Fig. 9) in combination with lower OPDA availability and support of OPDA biosynthesis (Fig. 7) antagonized trigger-induced gene regulation during the early stages of triggering.

### Conclusions

We conclude that oxylipin-controlled priming of genes for oxylipin biosynthetic enzymes and tAPX-related priming-regulation of *ZAT10* are parallel processes that are independently controlled by cold-priming and quickly affect trigger-induced regulation of gene expression. The tAPX-mediated pathway is specific for cold-priming-dependent regulation of *ZAT10* and co-regulates genes such as *ZAT6* and *ZAT12* upon cold-triggering. In contrast, oxylipin-priming is a more general mechanism that functions in the cold and in the light after cold-priming. The oxylipin effect shares similarities with processes activated by dehydration-priming and priming by high light (Liu *et al.*, 2016; Crisp *et al.*, 2017), pointing to the existence of a central and general priming mechanism.

## Supplementary data

The following supplementary data are available at *JXB* online.

Fig. S1. Genotyping and characterization of the *OPR3* T-DNA insertion line SALK_201355.

Fig. S2. Relative transcript accumulation for 20 of the genes identified by Park *et al.* (2015) as ‘first-wave’ cold-induced genes and represented with an FPKM higher than 0.5 in our data set as observed after 30 min of cold-triggering.

Fig. S3. Transcript abundances of stress-responsive and reference genes as determined by qPCR to confirm the reliability of the RNA-seq data.

Fig. S4. Cluster analysis of genes with at least 1.5-fold changes in expression during both cold- and light-triggering as a result of priming (CL-clusters).

Fig. S5. Cluster analysis of genes with at least 1.5-fold changes in expression during cold-triggering as a result of priming, with weaker effects of light-triggering (C-clusters).

Fig. S6: Cluster analysis of genes with at least 1.5-fold changes in expression during light-triggering as a result of priming, with weaker effects of cold-triggering (L-clusters).

Table S1. Primers used for genotyping the transgenic plant lines.

Table S2. Primers used for qPCR analyses.

Table S3. Read numbers and alignment efficiencies in the RNA-seq samples.

Table S4. FPKM values for the eight constitutively expressed genes as obtained in the RNA-seq analysis.

Table S5. Analysis of the data set for genes differentially expressed by cold- and light-triggering.

Table S6. FPKM values for the 1860 genes that passed all the pre-selection criteria and the impact of priming and cold- or light-triggering on their expression.

Table S7. JA- and OPDA-regulated genes according to Hickman *et al.* (2017) and Taki *et al.* (2005) in the groups of genes showing priming regulation by cold-triggering only, light-triggering only, and both cold- and light-triggering.

Table S8. SA-induced genes in the CL-clusters 4, 7, and 8 according to Blanco *et al.* (2009).

erab314_suppl_Supplementary_Figures_S1-S6

erab314_suppl_Supplementary_Tables_S1-S8

## Data Availability

All RNA-seq data are available at NCBI-GEO (GSE162507; GSM4953095–GSM4953107). All other data supporting the findings of this study are available within the paper and within its supplementary materials published online.

## Abbreviations

ABA: abscisic acid
AOC: allene oxide cyclase
AOS: allene oxide synthase
CBF: C-repeat binding factor
FPKM: fragments per kilobase of exon per million reads mapped
iOE: inducible overexpression
iRNAi: inducible RNAi
JA: jasmonic acid
JA-Ile: jasmonate conjugated with isoleucine
KO: knock-out
LOX: lipoxygenase
OPDA: 12-oxo phytenoic acid
OPR3: 12-oxophytodeinoate reductase 3
qPCR: quantitative PCR
RNA-seq: RNA-sequencing
SA: salicylic acid
tAPX: thylakoid-bound ascorbate peroxidase
T-DNA: transfer DNA
