# Supplementary material for: Cold-priming causes dampening of oxylipin biosynthesis and signalling during the early cold- and light-triggering response of Arabidopsis thaliana
Source: J Exp Bot. 2021 Jun 29;72(20):7163–79. doi: 10.1093/jxb/erab314 (PMC8547158; doi:10.1093/jxb/erab314)
Supplement: erab314_suppl_Supplementary_Figures_S1-S6 [file erab314_suppl_supplementary_figures_s1-s6.pdf]

Cold-priming causes dampening of oxylipin biosynthesis and signalling during the early cold- and light-triggering response of *Arabidopsis thaliana*

Andras Bittner, Bettina Hause and Margarete Baier

Suppl. Fig. S1

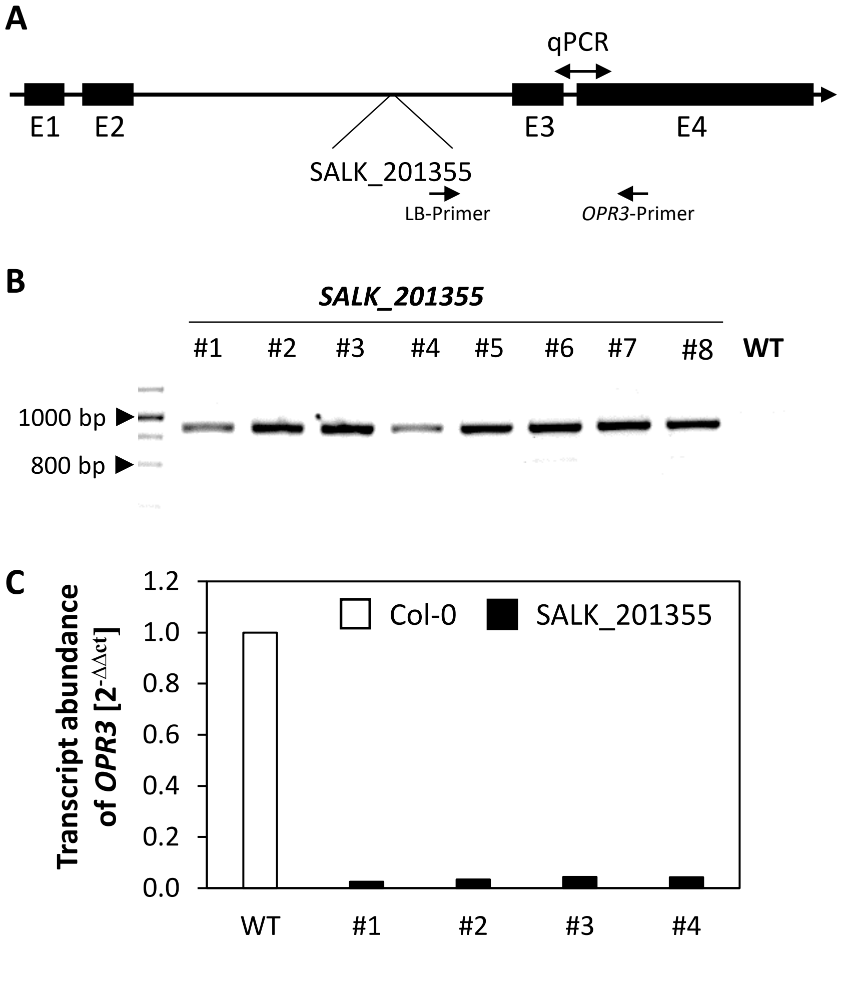

Suppl. Fig. S1: Genotyping and characterization of the *OPR3-T-DNA* insertion line SALK_201355: A) The T-DNA is inserted in the intron between exon 2 and exon 3. The black arrows show the primer position for qPCR-based *OPR3* transcript analysis and for PCR-based genotyping with an *OPR3*-specific and the T-DNA-specific left border primer (LB). **B)** For identity checking, PCR reaction were performed with the T-DNA-specific left-border primer (LB) and the gene-specific *OPR3*-primer and DNA of 8 independent SALK_201355 plants and one wild type Col-0 plant. The mutant allele gave the expected product of 900 base pairs (bp). **C)** The transcript abundance of *OPR3* was analysed in Col-0 (grey) and four homozygote SALK_201355 plants (green) by quantitative qPCR analysis. The transcript levels were normalized against the transcript abundance of the reference gene *YLS8*.

Cold-priming causes dampening of oxylipin biosynthesis and signalling during the early cold- and light-triggering response of *Arabidopsis thaliana*

Andras Bittner, Bettina Hause and Margarete Baier

Suppl. Fig. S2

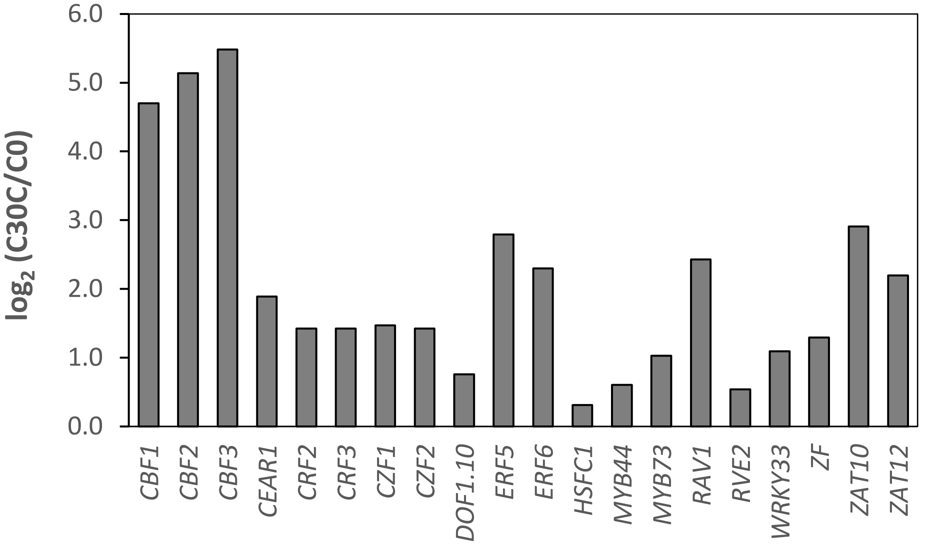

Suppl. Fig. S2: Relative transcript accumulation for 20 of the genes identified by Park *et al.* (2015) as 'first-wave' cold-induced genes and represented with an FPKM higher than 0.5 in our data set as observed after 30 min of cold-triggering.

Cold-priming causes dampening of oxylipin biosynthesis and signalling during the early cold- and light-triggering response of *Arabidopsis thaliana*

Andras Bittner, Bettina Hause and Margarete Baier

Suppl. Fig. S3

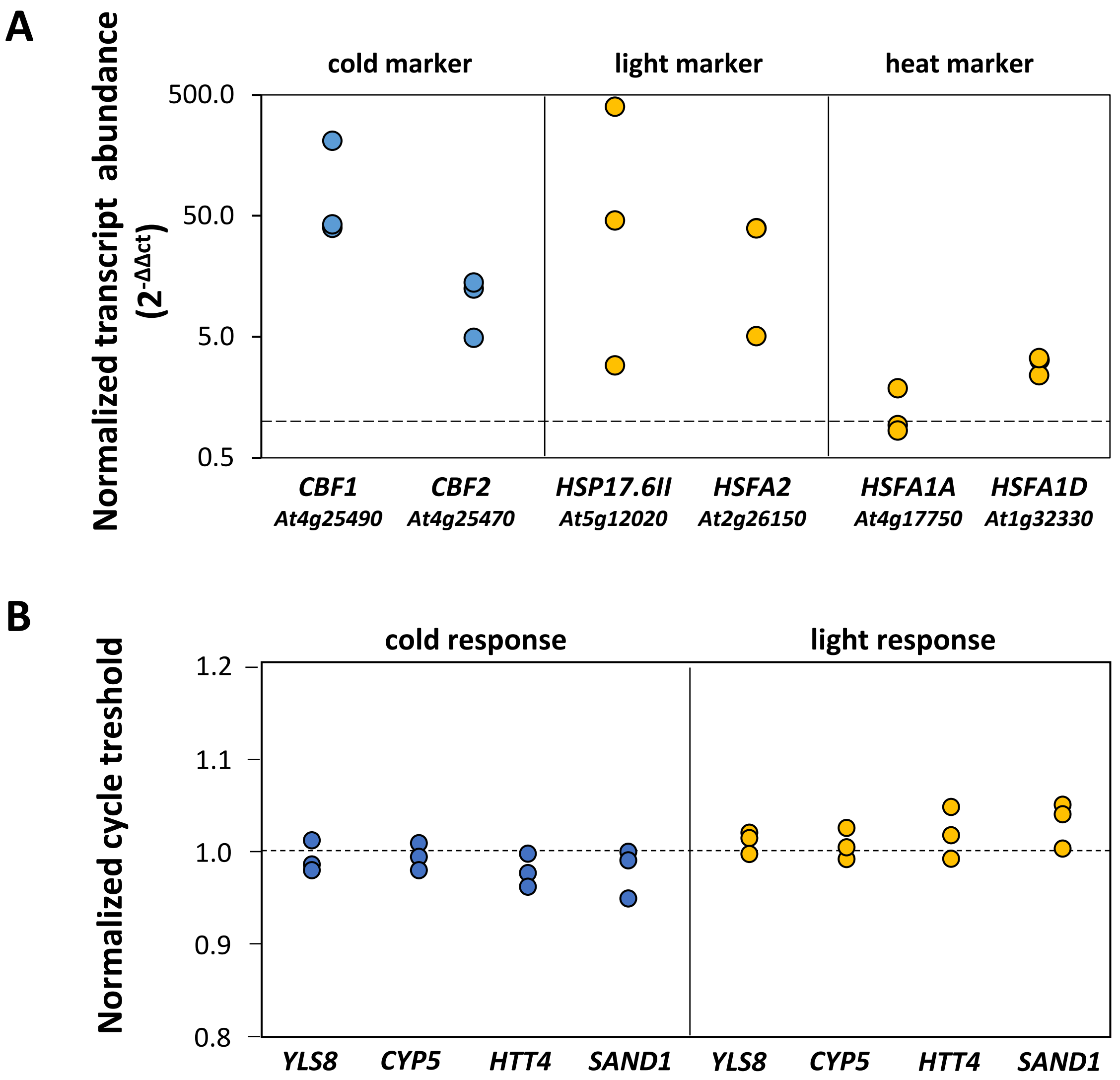

Suppl. Fig. S3: Transcript abundances of stress-responsive and reference genes as determined by qPCR to confirm the reliability of the RNA-seq data: A) Transcript abundances of the cold responsive genes *CBF1* and *CBF2*, the excess light responsive genes *HSP17.6II* and *HSFA2* and of the non-heat filtered light responsive genes were analysed in four-weak-old plants, which were either exposed for 60 min to 4°C (blue) or to heat-filtered excess light (800 µmol m^{-2} s^{-1}, yellow circle). The transcript values were normalized on the transcript abundances of *YLS8* and standardized to the transcript abundances in control plants (n=3) **B)** Transcript abundances of four reference genes, namely *YLS8*, CYP5, *HTT4* and *SAND1*, were analysed in untreated control plants and after 60 min in the cold or in high-light. The transcript abundances are shown as cycle-thresholds relative to the control cycle threshold (n=3).

Cold-priming causes dampening of oxylipin biosynthesis and signalling during the early cold- and light-triggering response of *Arabidopsis thaliana*

Andras Bittner[1], Bettina Hause[2] and Margarete Baier[1]*

Suppl. Fig. S4A

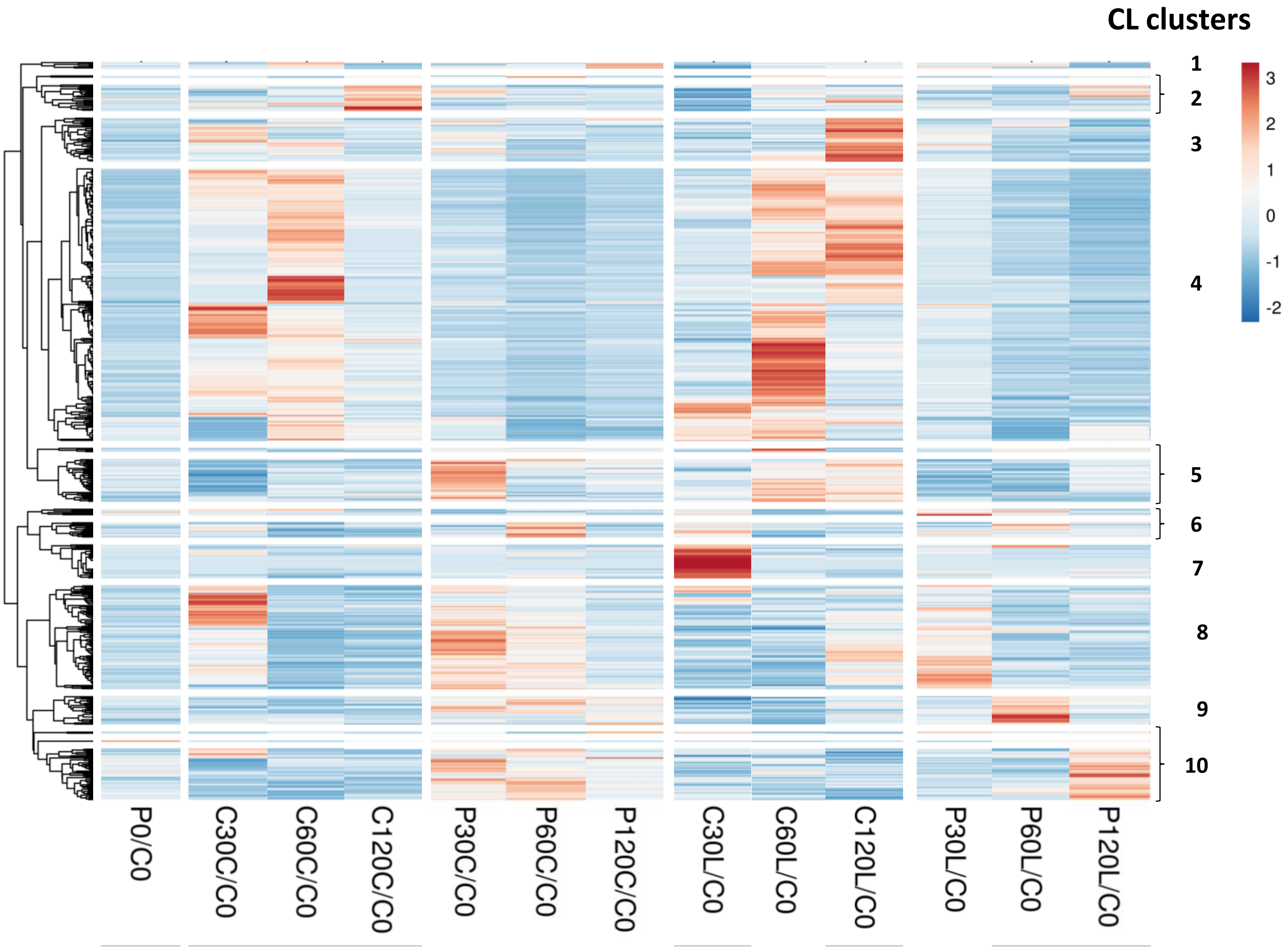

Suppl. Fig. 4A: Cluster analysis of genes with at least 1.5-fold priming effects in the cold and in the light. *Mean-centered heatmap and cluster-definition as obtained by ClustVis with the C0-normalized FPKM data listed in Suppl. Tab. S9.*

Cold-priming causes dampening of oxylipin biosynthesis and signalling during the early cold- and light-triggering response of *Arabidopsis thaliana*

Andras Bittner[1], Bettina Hause[2] and Margarete Baier[1]*

Suppl. Fig. S4B

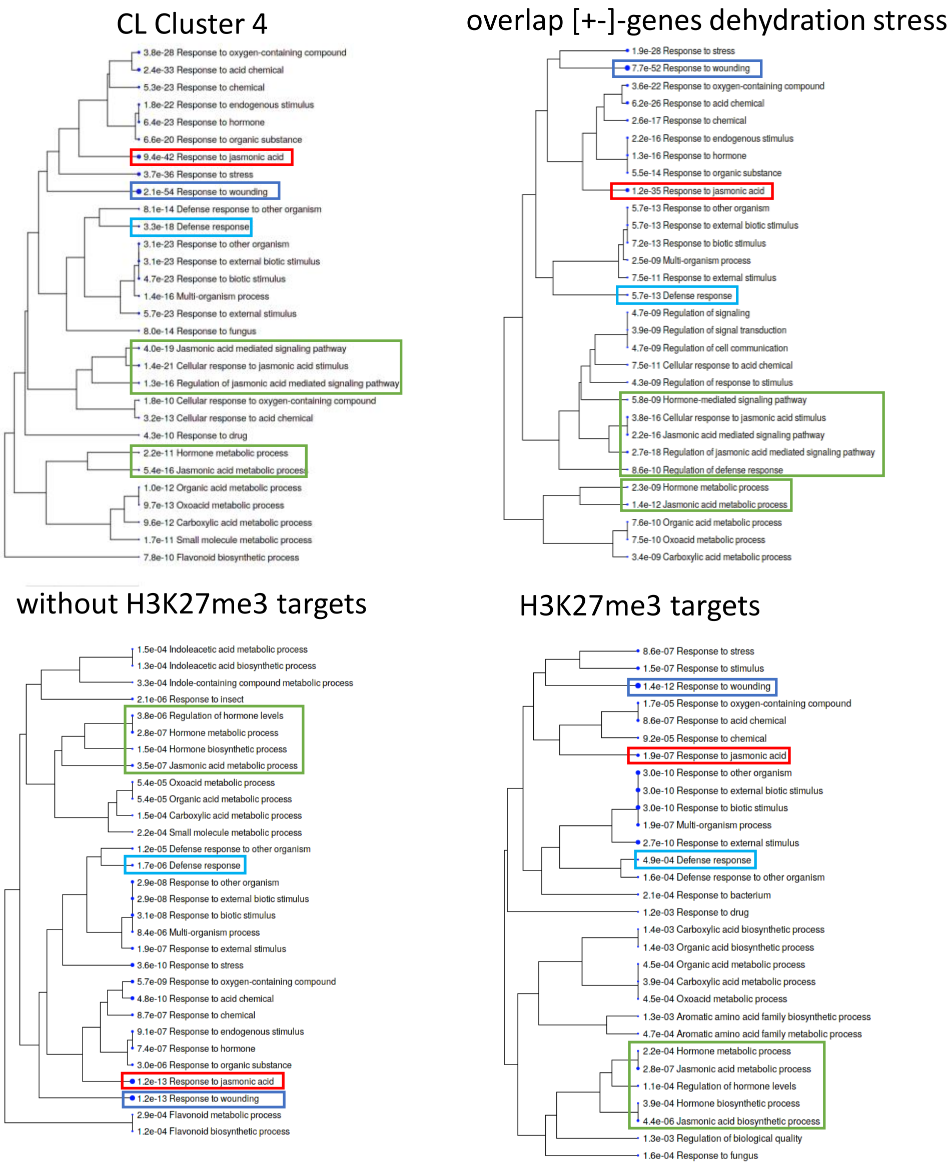

Suppl. Fig. 4B: *Results on GO-term analysis for functional categories on the CL cluster 4: all genes (top left), genes overlapping with the [+ -] cluster of dehydration priming-responsive genes (top right), CL cluster 4 genes with are no H3K27me3 targets (bottom left) and the H3K27me3 target genes of CL cluster 4.*

Cold-priming causes dampening of oxylipin biosynthesis and signalling during the early cold- and light-triggering response of *Arabidopsis thaliana*

Andras Bittner[1], Bettina Hause[2] and Margarete Baier[1]*

Suppl. Fig. S4C

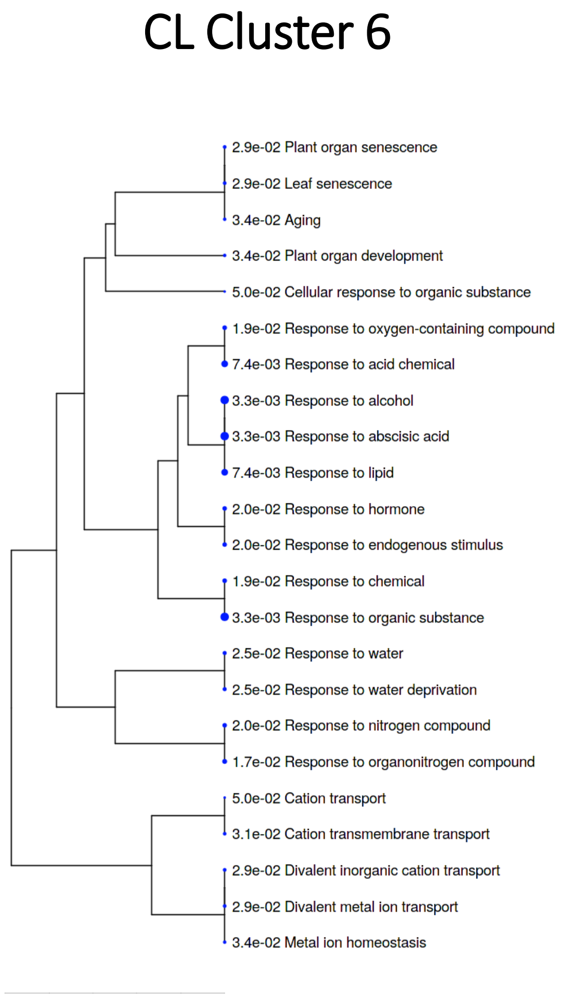

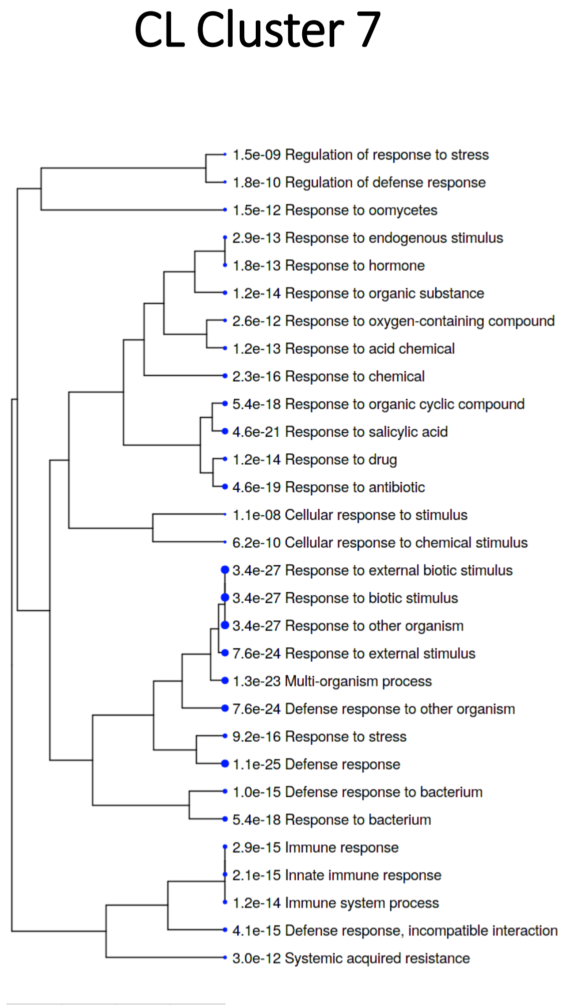

Suppl. Fig. S4C: *Results on GO-term analysis for functional categories on the CL cluster 6 and 7.*

Cold-priming causes dampening of oxylipin biosynthesis and signalling during the early cold- and light-triggering response of *Arabidopsis thaliana*

Andras Bittner[1], Bettina Hause[2] and Margarete Baier[1]*

Suppl. Fig. S4D

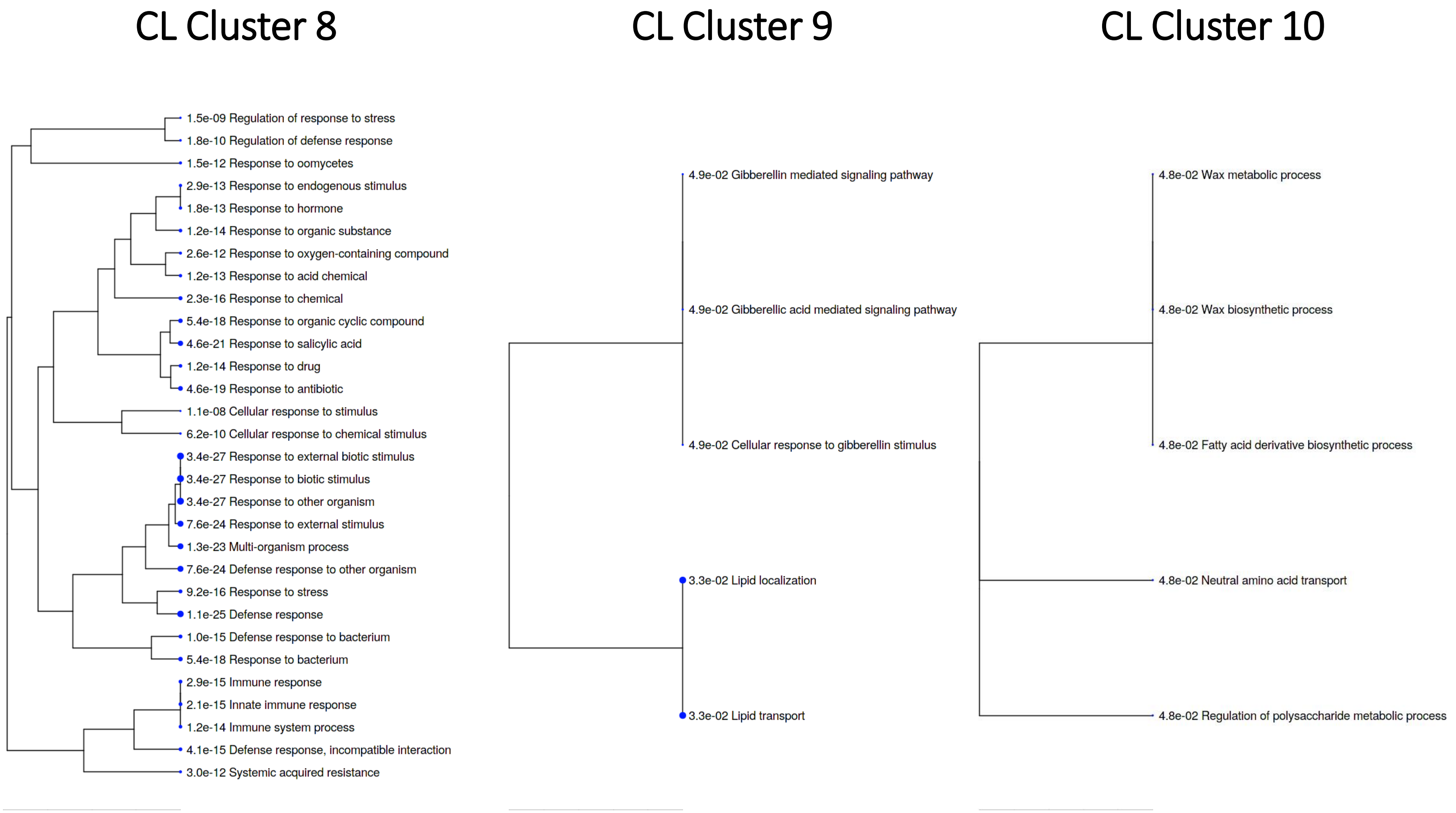

Suppl. Fig. S4D: *Results on GO-term analysis for functional categories on the CL clusters 8, 9 and 10.*

Cold-priming causes dampening of oxylipin biosynthesis and signalling during the early cold- and light-triggering response of *Arabidopsis thaliana*

Andras Bittner[1], Bettina Hause[2] and Margarete Baier[1]*

Suppl. Fig. S5A

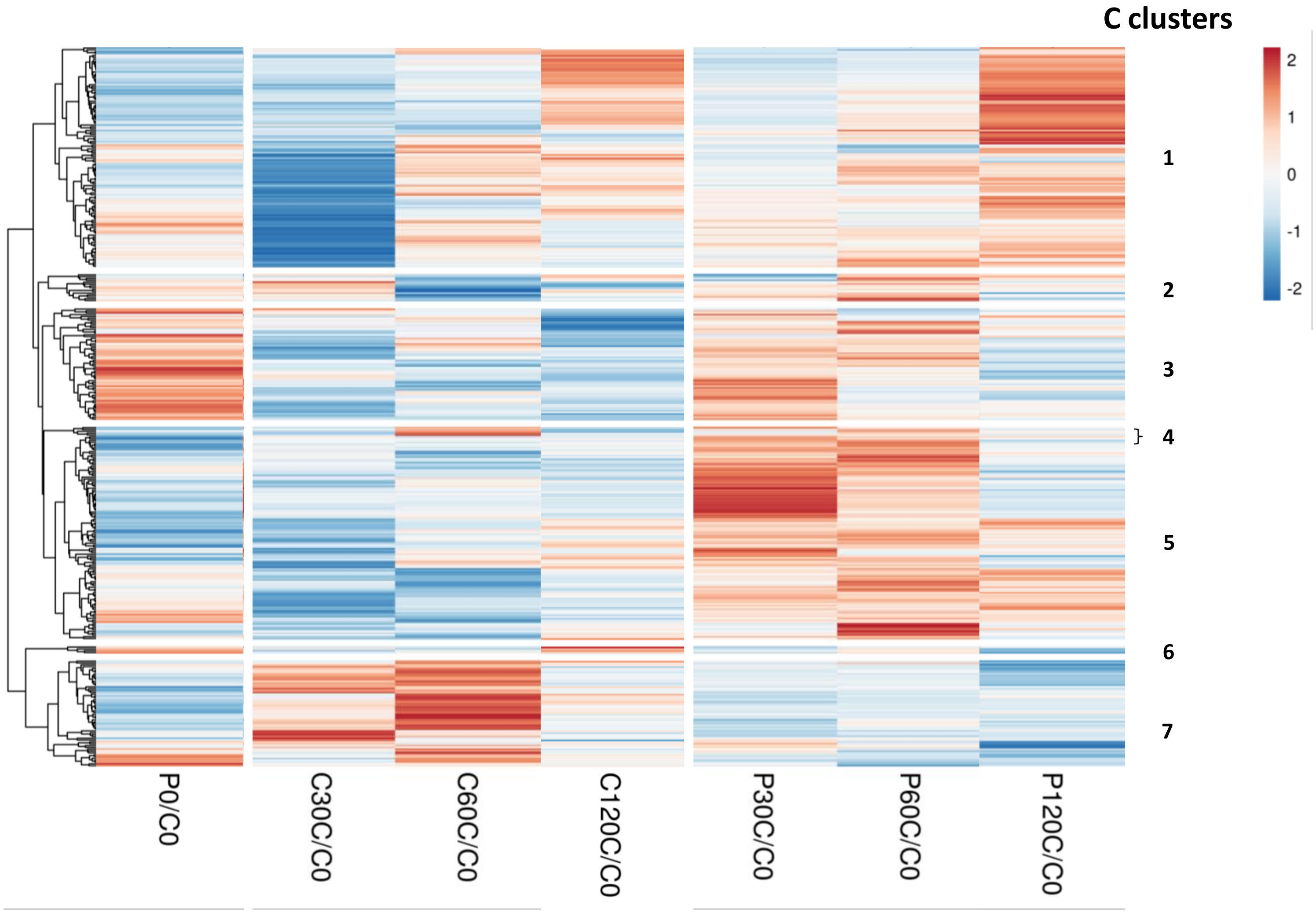

Suppl. Fig. S5A: Cluster analysis of genes with at least 1.5-fold priming effects in the cold and weaker priming effects in the light. *Mean-centered heatmap and cluster-definition as obtained by ClustVis with the C0-normalized FPKM data listed in Suppl. Tab. S7.*

Cold-priming causes dampening of oxylipin biosynthesis and signalling during the early cold- and light-triggering response of *Arabidopsis thaliana*

Andras Bittner[1], Bettina Hause[2] and Margarete Baier[1]*

Suppl. Fig. S5B

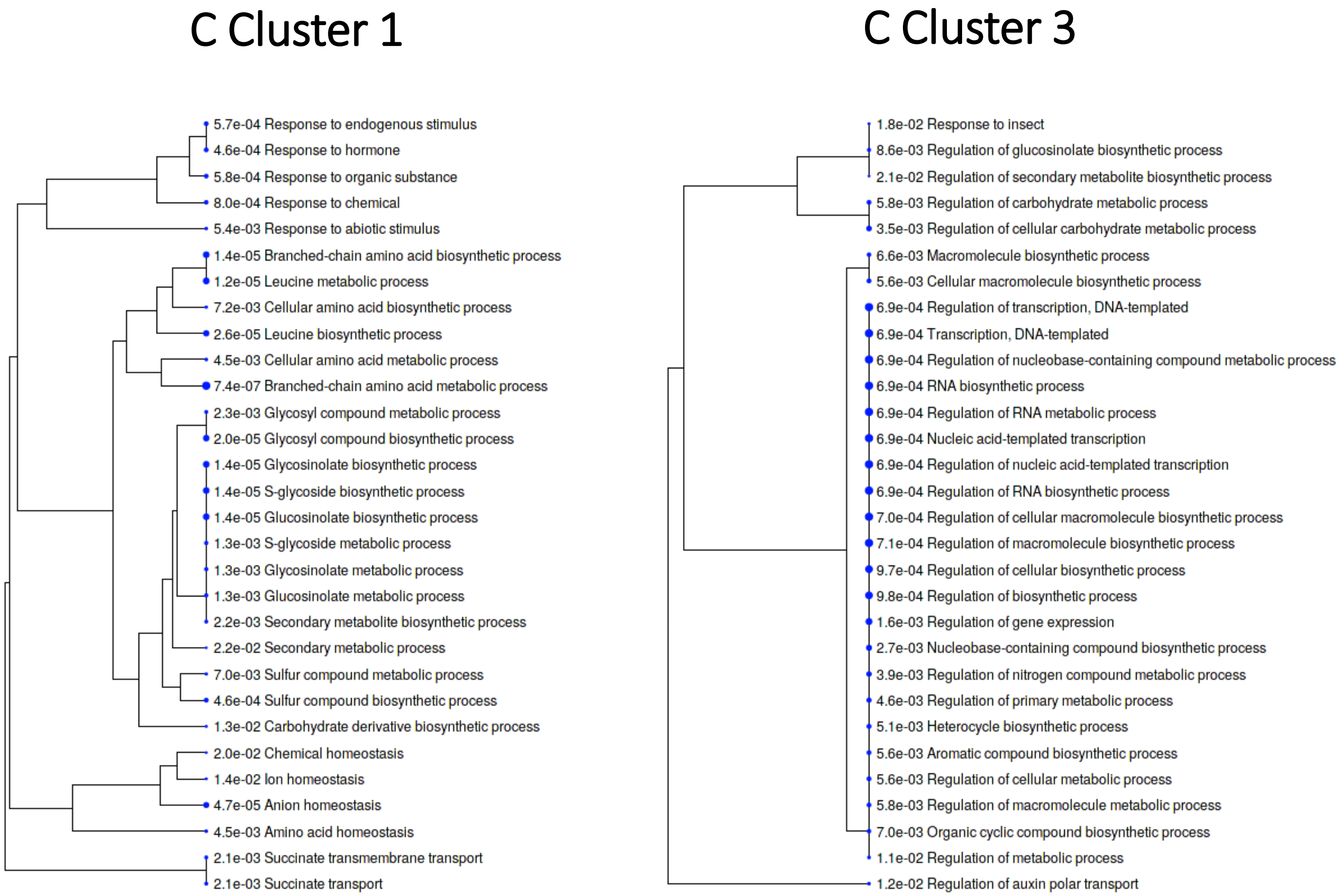

Suppl. Fig. S6B: *Results on GO-term analysis for functional categories on the C cluster 1 and 3.*

Cold-priming causes dampening of oxylipin biosynthesis and signalling during the early cold- and light-triggering response of *Arabidopsis thaliana*

Andras Bittner[1], Bettina Hause[2] and Margarete Baier[1]*

Suppl. Fig. S5C

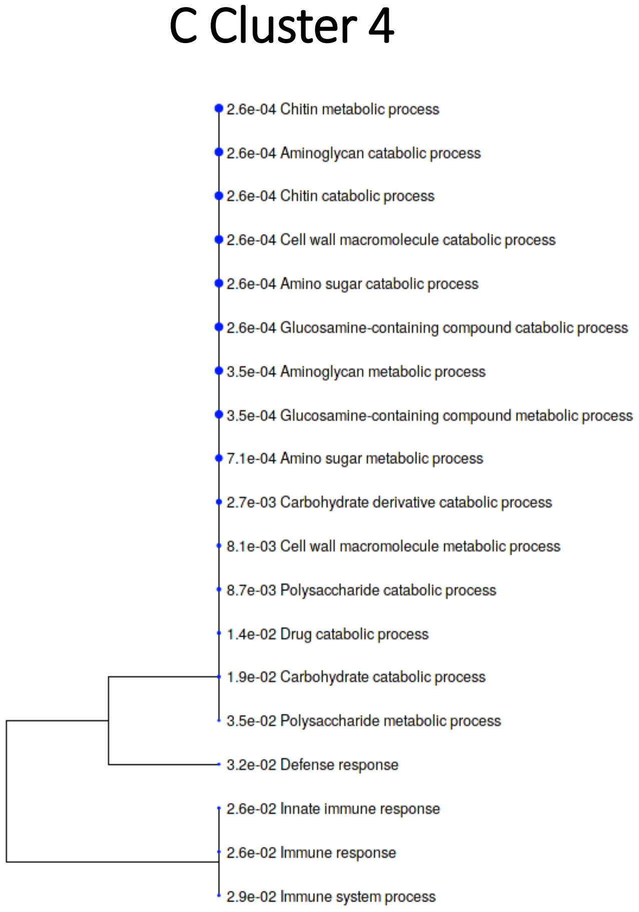

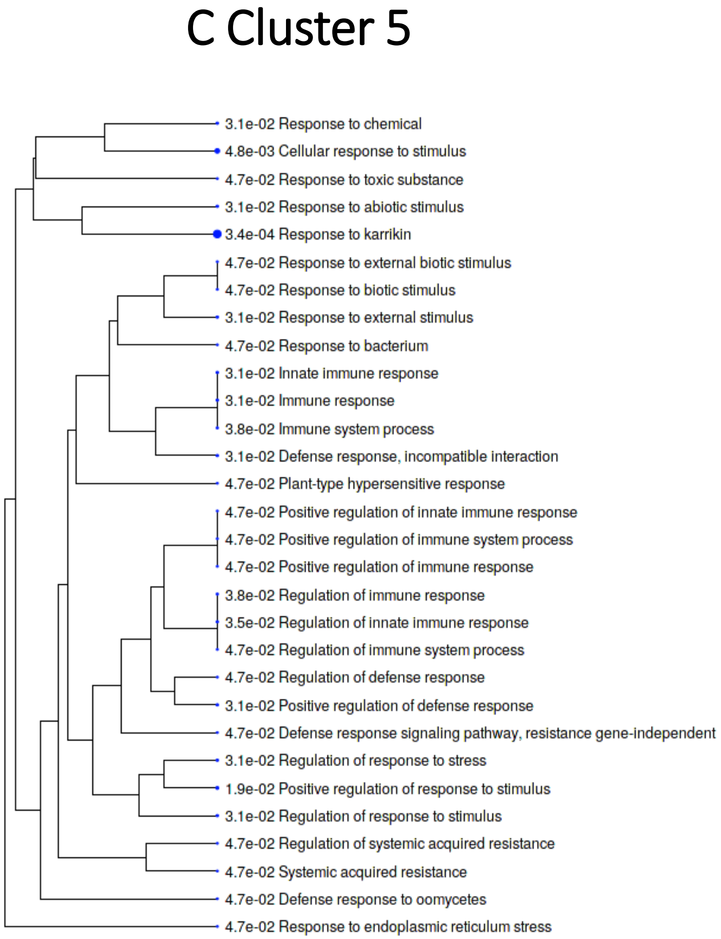

Suppl. Fig. S5C: *Results on GO-term analysis for functional categories on the C cluster 4 and 5.*

Cold-priming causes dampening of oxylipin biosynthesis and signalling during the early cold- and light-triggering response of *Arabidopsis thaliana*

Andras Bittner[1], Bettina Hause[2] and Margarete Baier[1]*

Suppl. Fig. S5D

C Cluster 6

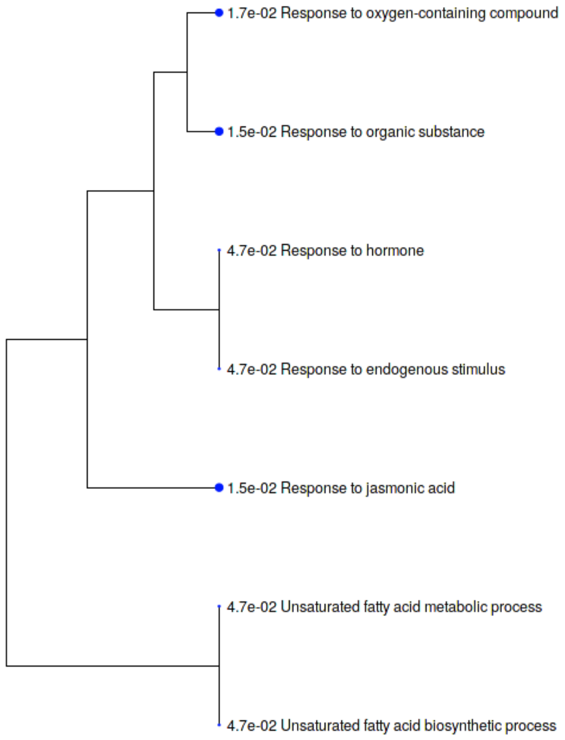

Suppl. Fig. S5D: *Results on GO-term analysis for functional categories on the C cluster 6.*

Cold-priming causes dampening of oxylipin biosynthesis and signalling during the early cold- and light-triggering response of *Arabidopsis thaliana*

Andras Bittner[1], Bettina Hause[2] and Margarete Baier[1]*

Suppl. Fig. S6A

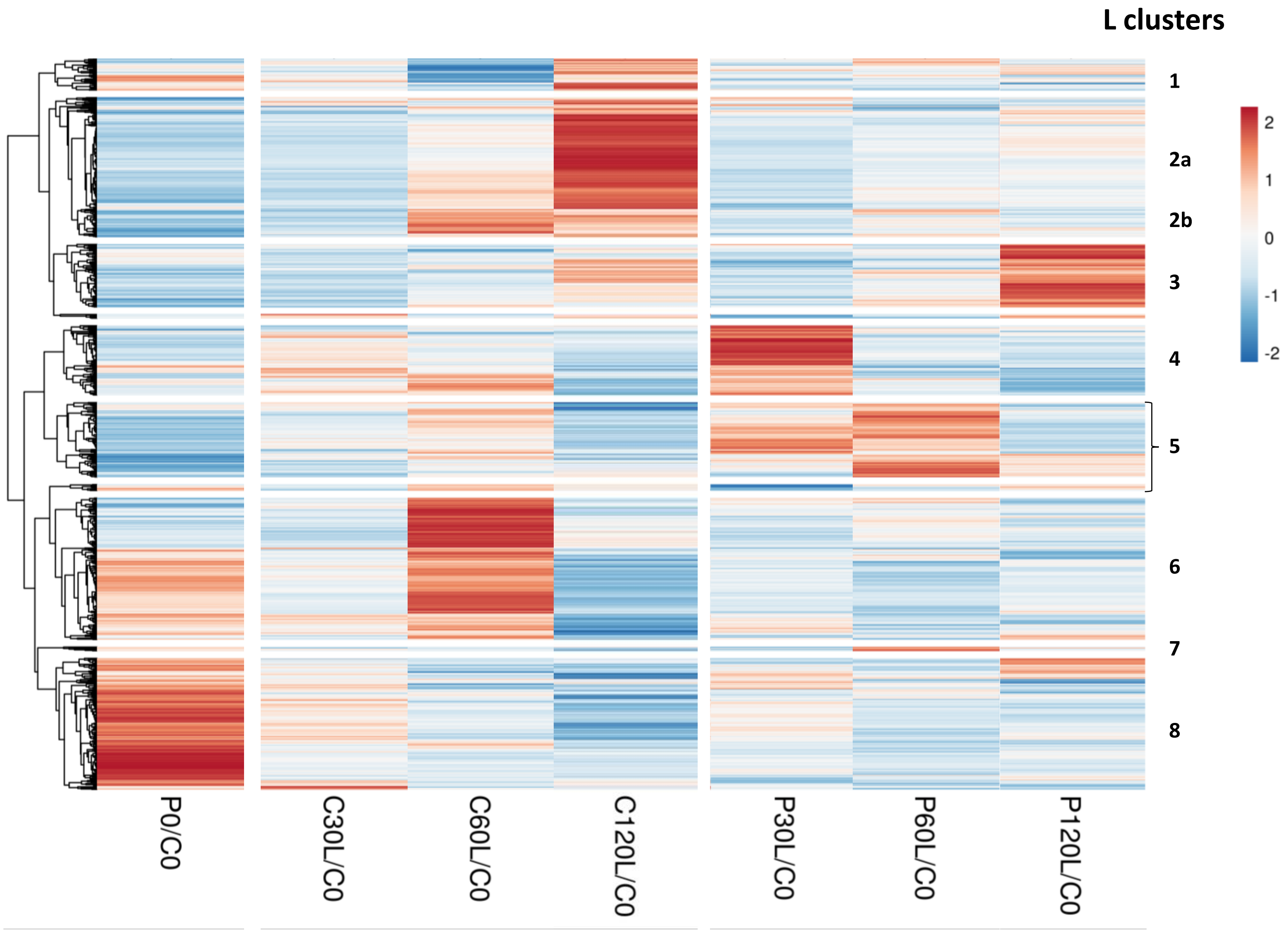

Suppl. Fig. S6A: Cluster analysis of genes with at least 1.5-fold priming effects in the light and weaker priming effects in the cold. *Mean-centered heatmap and cluster-definition as obtained by ClustVis with the C0-normalized FPKM data listed in Suppl. Tab. S8.*

Cold-priming causes dampening of oxylipin biosynthesis and signalling during the early cold- and light-triggering response of *Arabidopsis thaliana*

Andras Bittner[1], Bettina Hause[2] and Margarete Baier[1]*

Suppl. Fig. S6B

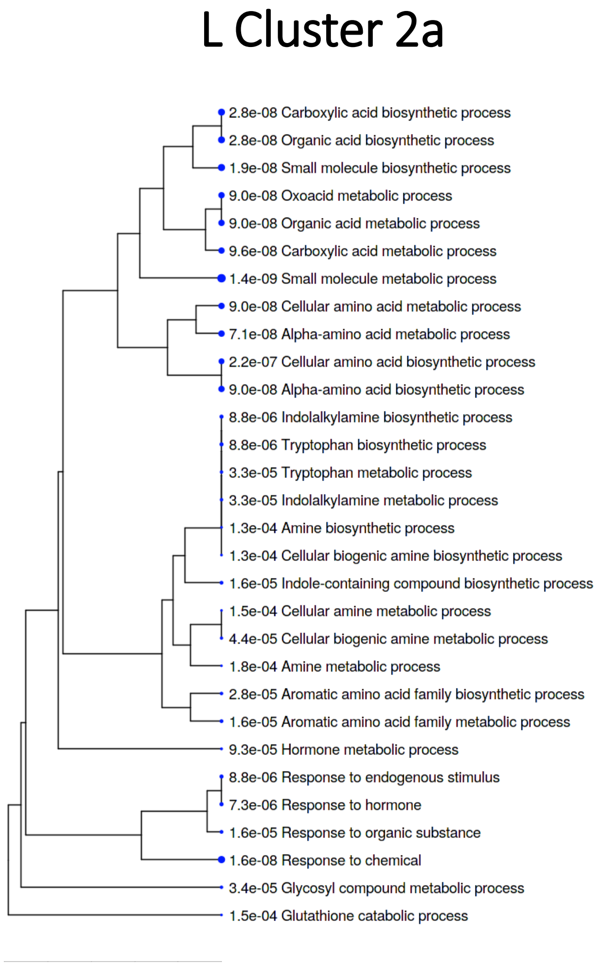

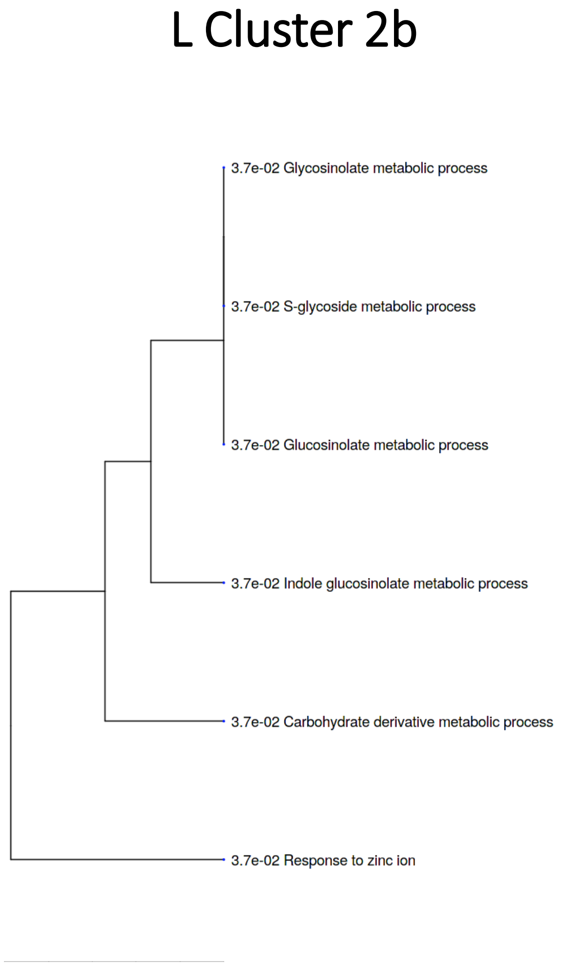

Suppl. Fig. S6B: *Results on GO-term analysis for functional categories on the L cluster 2a and 2b.*

Cold-priming causes dampening of oxylipin biosynthesis and signalling during the early cold- and light-triggering response of *Arabidopsis thaliana*

Andras Bittner[1], Bettina Hause[2] and Margarete Baier[1]*

Suppl. Fig. S6C

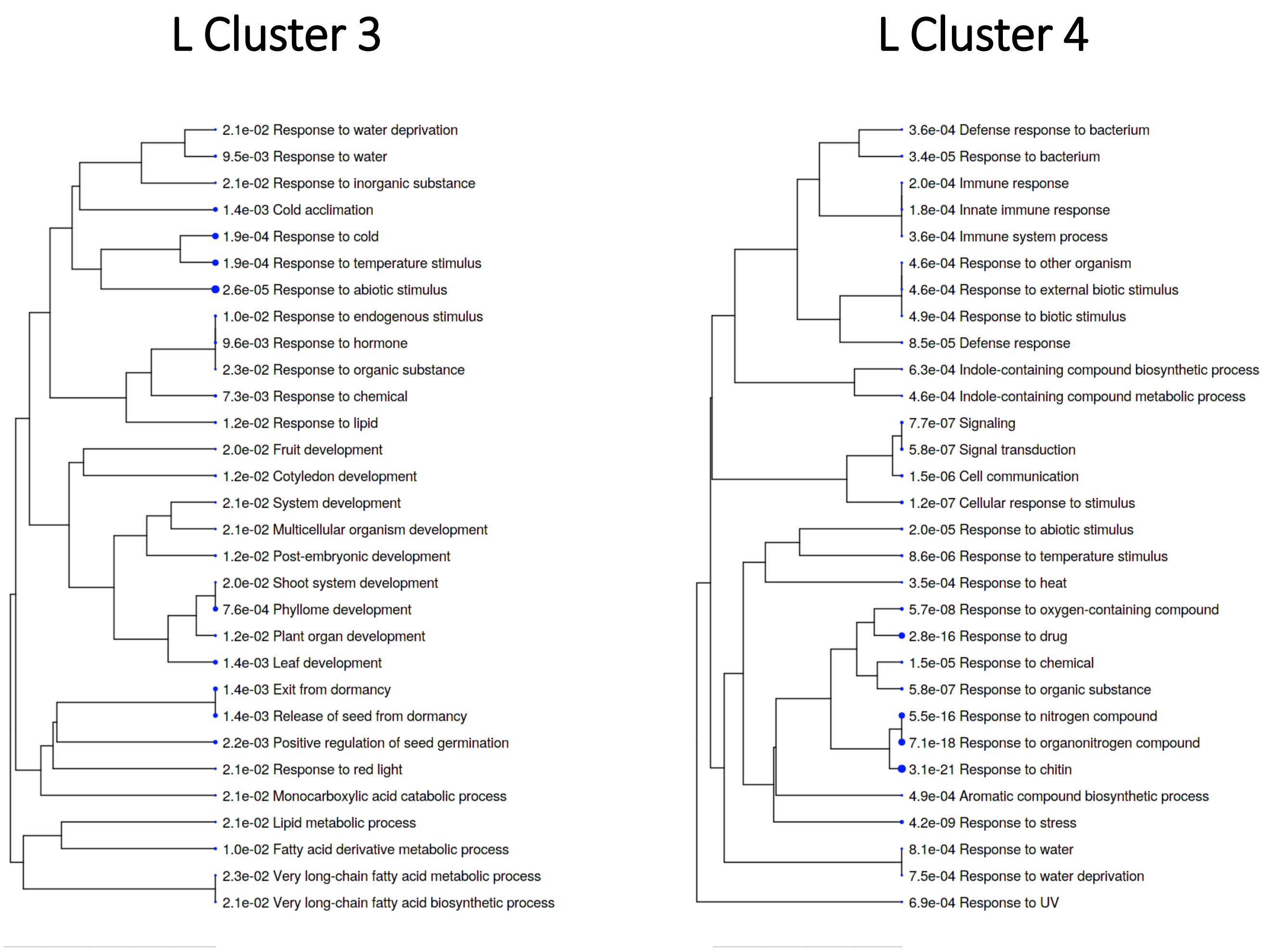

Suppl. Fig. S6C: *Results on GO-term analysis for functional categories on the L cluster 3 and 4.*

Cold-priming causes dampening of oxylipin biosynthesis and signalling during the early cold- and light-triggering response of *Arabidopsis thaliana*

Andras Bittner[1], Bettina Hause[2] and Margarete Baier[1]*

Suppl. Fig. S6D

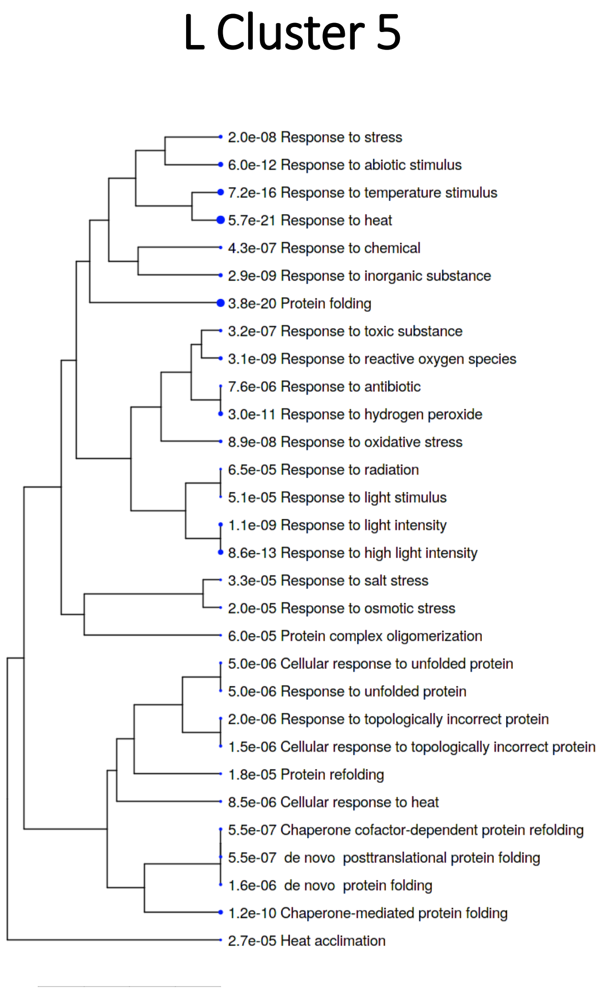

Suppl. Fig. S6D: *Results on GO-term analysis for functional categories on the L clusters 5 and 6.*
